# Cytotoxicity of *Prymnesium parvum* extracts and prymnesin analogs on epithelial fish gill cells RTgill-W1 and the human colon cell line HCEC-1CT

**DOI:** 10.1007/s00204-023-03663-5

**Published:** 2024-01-11

**Authors:** Elisabeth Varga, Hélène-Christine Prause, Matthias Riepl, Nadine Hochmayr, Deniz Berk, Eva Attakpah, Endre Kiss, Nikola Medić, Giorgia Del Favero, Thomas Ostenfeld Larsen, Per Juel Hansen, Doris Marko

**Affiliations:** 1 Department of Food Chemistry and Toxicology, Faculty of Chemistry, University of Vienna, Währinger Str. 38-40, 1090 Vienna, Austria; 2 Unit Food Hygiene and Technology, Institute of Food Safety, Food Technology and Veterinary Public Health, University of Veterinary Medicine, Vienna, Veterinärplatz 1, 1210 Vienna, Austria; 3 Vienna Doctoral School in Chemistry, Faculty of Chemistry, University of Vienna, Währinger Str. 42, 1090 Vienna, Austria; 4 Core Facility Multimodal Imaging, Faculty of Chemistry, University of Vienna, Währinger Str. 38-42, 1090 Vienna, Austria; 5 Marine Biological Section, Department of Biology, University of Copenhagen, Strandpromenaden 5, 3000 Helsingør, Denmark; 6 Center for Bioresources, Division for Food and Production, Danish Technological Institute, Gregersensvej 8, 2630 Taastrup, Denmark; 7 Department of Biotechnology and Biomedicine, Technical University of Denmark, Søltofts Plads 221, 2800 Kgs Lyngby, Denmark

**Keywords:** Microalgae, Ichthyotoxin, UHPLC, MS, Fluorescence microscopy, Ionoregulation

## Abstract

Harmful algal blooms kill fish populations worldwide, as exemplified by the haptophyte microalga *Prymnesium parvum*. The suspected causative agents are prymnesins, categorized as A-, B-, and C-types based on backbone carbon atoms. Impacts of *P. parvum* extracts and purified prymnesins were tested on the epithelial rainbow trout fish gill cell line RTgill-W1 and on the human colon epithelial cells HCEC-1CT. Cytotoxic potencies ranked A > C > B-type with concentrations spanning from low (A- and C-type) to middle (B-type) nM ranges. Although RTgill-W1 cells were about twofold more sensitive than HCEC-1CT, the cytotoxicity of prymnesins is not limited to fish gills. Both cell lines responded rapidly to prymnesins; with EC_50_ values for B-types in RTgill-W1 cells of 110 ± 11 nM and 41.5 ± 0.6 nM after incubations times of 3 and 24 h. Results of fluorescence imaging and measured lytic effects suggest plasma membrane interactions. Postulating an osmotic imbalance as mechanisms of toxicity, incubations with prymnesins in media lacking either Cl^−^, Na^+^, or Ca^2+^ were performed. Cl^−^ removal reduced morphometric rearrangements observed in RTgill-W1 and cytotoxicity in HCEC-1CT cells. Ca^2+^-free medium in RTgill-W1 cells exacerbated effects on the cell nuclei. Prymnesin composition of different *P. parvum* strains showed that analog composition within one type scarcely influenced the cytotoxic potential, while analog type potentially dictate potency. Overall, A-type prymnesins were the most potent ones in both cell lines followed by the C-types, and lastly B-types. Disturbance of Ca^2+^ and Cl^−^ ionoregulation may be integral to prymnesin toxicity.

**Electronic supplementary material:**

The online version of this article (10.1007/s00204-023-03663-5) contains supplementary material, which is available to authorized users.

## Introduction

Microalgae play an important role in the marine ecosystem, i.e., the production of oxygen, uptake of carbon dioxide (CO_2_) and inorganic nutrients and provide food for the entire aquatic food web (Tsai et al. 2012). In general, they can be divided into macroalgae, which are visible to the naked eye, and microalgae, which are one-cell organisms and can be of prokaryotic or eukaryotic nature. Microalgae can on the one hand produce nutrient-rich and beneficial biomolecules like fatty acids, lipids, and vitamins, and on the other hand, produce harmful toxins. Among the most important harmful algal bloom (HAB)-forming microalga worldwide is the haptophyte *Prymnesium parvum* (Hallegraeff 1993; Manning and La Claire 2010). These HABs are characterized by a rapid proliferation of the microalgae, and can have huge economic and ecological impacts, lasting from several days to months, depending on the given environmental conditions (Ryan et al. 2017; Vasas et al. 2012). HABs can lead to massive fish kills (Manning and La Claire 2010) by lowering oxygen levels in the water, damaging the inhabitants of the marine life by toxin production or causing direct damage to fish gills as well as clogging (Hallett et al. 2016). Just last summer (August 2022), 360 t of fish died in the Oder River in Poland/Germany due to this alga (Free et al. 2023).

**Fig. 1:**
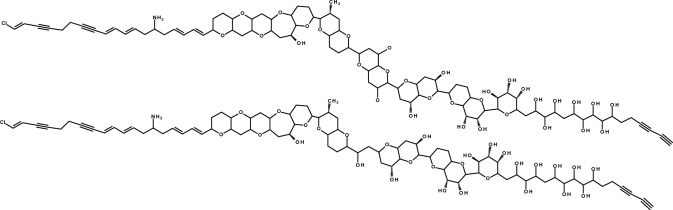
Backbone structure of **A**- (top) and **B**-type (bottom) prymnesins according to Igarashi et al. (1996) and Rasmussen et al. (2016a), respectively. The main difference is the length of the carbon backbone, further modifications of prymnesin analogs are attached sugar moieties, the degree of saturation and the number of incorporated chloride and oxygen atoms

In case of *P. parvum*, the prymnesins (PRMs) have been identified as causative ichthyotoxic agents and possess cytotoxic and hemolytic properties (Binzer et al. 2019; Igarashi et al. 1996). The height of the blooms often occurs when the environmental conditions are suboptimal (Granéli and Salomon 2010; Manning and La Claire 2010; Shilo 1967; Svenssen et al. 2019). PRMs are supersized (1600–2300 Da) ladder-frame polyethers (Fig. 1), currently grouped into three categories A-, B-, and C-type PRMs (Igarashi et al. 1996, 1999; Rasmussen et al. 2016b). They differ in the length of their aglycon backbone, the longest being A-type with 91, followed by B- and C-type, with 85 and 83 carbon atoms, respectively (Binzer et al. 2019). While the backbone structure of two A- (prymnesin-1 and prymnesin-2, Igarashi et al. 1996, 1999) and one B-type (prymnesin B1, Rasmussen et al. 2016a) were fully characterized by NMR, the exact structure of C-type PRMs remains unclear. Within each category exists a large diversity in terms of degree of saturation, chlorination, and attached sugar moieties (Binzer et al. 2019). One feature the three groups share is a primary amine located in the lipophilic part of the ladder-frame (Fig. 1) (Igarashi et al. 1996, 1999; Rasmussen et al. 2016a). One strain of *P. parvum* produces exclusively one type of toxin, but different analogs thereof, and the toxic potential varies between the types (Binzer et al. 2019; Rasmussen et al. 2016a). Overall, identifying the strain and thus category of PRM produced is essential for understanding *P. parvum* toxicity. Whether the type of PRM analog influences the potency, however, remains unclear.

Fish mortality due to these toxins is caused by gill damage, typically manifesting as increased permeability, ultimately leading to internal oxygen deficiency (Svendsen et al. 2018; Ulitzur and Shilo 1966; Yariv and Hestrin 1961). This is seemingly achieved through pore formation in gill cells, resulting in disruption of the ionic balance and increased mucus production (Otterstrom and Nielsen 1939; Ulitzur and Shilo 1964, 1966; Tillmann 2003). When investigating HAB-forming microalgae, scientists typically resort to fish bioassays for acute or sublethal toxicity in brine shrimp, larval or juvenile fish (Segner 1998; Svendsen et al. 2018; McKim et al. 1987). These bioassays have been widely criticized especially for ethical reasons, given the large number of animals that are required, and the severe harm imposed on them. Also, acute fish toxicity assays only reflect a relative toxicity where no specific mode-of action is investigated (Fischer et al. 2019; Segner et al. 1998). The use of cell lines such as the epithelial rainbow trout (*Oncorhynchus mykiss* (Walbaum)) fish gill cell line RTgill-W1 as an alternative represents a target-specific test system, that does not require the sacrifice of live animals (Bols et al. 1994; Segner 1998). By the same token, it allows for the investigation of the mode of action of ichthyotoxins, with the possibility to select various endpoints (Fischer et al. 2019; Segner 1998). Since June 2021, a guideline for testing the acute toxicity of chemicals with the RTgill-W1 cell line is available from the Organisation for Economic Co-operation and Development (OECD 2021).

The exact mechanism through which PRMs exhibit their toxicity remains unclear, although evidence points to a direct interaction with the cell membrane (Igarashi et al. 1998; Shilo 1967). Brevetoxins which share a similar ladder-frame core structure as PRM were found to bind to voltage-sensitive Na^+^-channels. A similar mode of action was hypothesized for PRMs (Baden 1989; Rasmussen et al. 2016b). Moreover, the presence or absence of ions in the medium has been shown to affect PRM toxicity in fish, further hinting at the involvement of ions (Shilo 1967; Ulitzur and Shilo 1966; Yariv and Hestrin 1961). PRM toxicity was observed also in short-term exposure of fish, which indicates a fast interaction with their physiological target (Svendsen et al. 2018).

Given the current state of knowledge, the aim of this study was to assess the toxic potential of A-, B-, and C-type PRMs. We hypothesize that different prymnesin types and different prymnesin analogs influence toxicity. To provide answers cell viability and membrane integrity of RTgill-W1 cells and the human epithelial colon cell line HCEC-1CT were assessed. The HCEC-1CT cell line was selected, because it cannot be excluded that humans will be exposed to PRMs. Other phycotoxins, such as brevetoxin aerosols, are known to have adverse effects on humans (Benson et al. 1999; Hallegraeff 1993; Sanseverino et al. 2016). Furthermore, a better understanding may be gained from comparing the response of cell lines derived from different species to PRM cytotoxicity. For evaluating the rapidity with which PRMs act, toxicity tests were performed for two incubation times (3 h vs. 24 h). Furthermore, we hypothesize that PRMs interact with ion channels and ionoregulation is a relevant factor for toxicity. Morphological changes upon exposure to PRMs were captured using fluorescent dyes during live-cell imaging. Additionally, the effect selected ions may have on the potency of PRMs was investigated and a possible influence of two RTgill-W1 media on the cell viability and morphology after exposure to PRMs was assessed.

## Materials and methods

### Chemicals and reagents

HPLC-grade acetone, methanol and acetonitrile (ACN) as well as absolute ethanol (99.8%) were purchased at Fisher Chemical (Loughborough, UK). HPLC grade formic acid was obtained from Carl Roth GmbH + Co. KG (Karlsruhe, Germany) and LC–MS grade water was from VWR (Vienna, Austria).

### Samples

The non-axenic microalgal strains were previously obtained from the Scandinavian Culture Collection of Algae and Protozoa (SCCAP, now incorporated in the Norwegian Culture Collection of Algae, NORCCA, strain K-0081), the Roscoff Culture Collection (RCC, strain RCC-191 and RCC-1436) or the Culture Collection of Algae of the University of Texas (UTEX, strain UTEX-2797). The strains were maintained in F/2 growth medium prepared from filtered (Whatman^®^ glass microfiber filters, grade GF/F, Sigma Aldrich, St. Louis, MO/USA) and pasteurized (95 °C, 95 min) natural seawater off the coast of Helsingør at around 30 salinity (Guillard 1975). They were kept at 15 °C and an irradiance of 450–500 µmol photons/(m^2^ s) on a 14:10 h light–dark cycle. *P. parvum* cultures used for the bioassays were inoculated and kept in exponential growth in 10 L glass bottles with gentle aeration to prevent elevation of pH due to photosynthesis. During the cultivation period, the cell concentrations were determined every 2–3 days using a flow cytometer (CytoFLEX flow cytometer, Beckman Coulter, Copenhagen, Denmark). Cells were distinguished from the background using pigment fluorescence (PC5.5) and forward scatter (FSC). The microalgal biomass was harvested in the late exponential growth using the Avanti J-26 XP, JFC-Z continues centrifuge (Beckmann Coulter) with flow rates between 15 and 20 mL/min at 4 °C and 3500 rounds per minutes (rpm, ca. 1111 relative centrifugal force, rcf). The biomass was transferred into falcon tubes and centrifuged again at 4000 rcf for 15 min to discard as much supernatant as possible. The biomass pellets were stored at  – 80 °C until further extraction. The inorganic carbon in the culture was measured using the total organic carbon analyzer (TOC-L, Shimadzu Europe GmbH, Duisburg, Germany). The extraction of PRMs followed the procedure described in (Rasmussen et al. 2016a; Binzer et al. 2019). First, the biomass was thawed and centrifuged at 4000 rcf for 5 min using a Z326-K centrifuge (Hermle Labortechnik GmbH, Wehingen, Germany) and then the aqueous supernatant was removed. The algal biomass samples were extracted several times with ice-cold acetone to remove the chlorophyll followed by extractions with methanol to obtain the PRMs. In-between the extraction steps, the samples were centrifuged, and the extracts of the same solvent were combined. The solvent was removed by a rotavapor (R-114, Büchi Labortechnik AG, Flawil, Switzerland) or a CentriVap Benchtop Vacuum Concentrator coupled to a CentriVap Coldtrap (both Labconco Corporation, Kansas City, MO/USA). The samples were reconstituted in absolute ethanol, treated in the ultrasonic bath for several minutes, centrifuged again and the particle free supernatant was transferred to HPLC vials.

The two extracts from UTEX-2797 will henceforward be called A1 and A2, and B-type extracts, all from strain K-0081, as B1-B4. Two C-type PRM extracts, C1 and C2, from strains RCC-1436 and RCC-191, respectively, were tested as well. Single compound solutions were previously purified by means of liquid chromatography and are referred to as sA1, sB1 and sB2. One A-type solution (A3) which was previously available from Sigma Aldrich as lysis standard (No. P-1389), was provided by Tom Shier (Department of Medicinal Chemistry, College of Pharmacy, University of Minnesota, Minneapolis/MN, USA).

### Chemical analysis

#### Quantitative analysis

For cell-based assays, it was essential to have analytical information about the investigated *P. parvum* samples. Hence, PRM concentrations were semi-quantified via high-performance liquid chromatography using a fluorescence detector (HPLC-FLD), with an indirect method previously described by Svenssen et al. (2019) with slight modifications. Fluorescence derivatization of the primary amine of the PRMs was performed using the AccQ-Tag Fluor Reagent Kit from Waters Corporation (Milford/MA, USA). Due to the lack of standards, a mixture of fumonisins B_1_ and B_2_ (Romer Labs, Tulln, Austria) was used as external calibrant. The chromatographic separation was performed on a 1200 HPLC system (Agilent Technologies, Waldbronn, DE), using an Agilent Poroshell C18 column (2.1 × 50 mm 2.7 µm) at 40 °C and a flow rate of 0.4 mL/min. The eluents were water (eluent A) and ACN (eluent B) and both contained 0.1% formic acid. A linear gradient was applied starting with 20% B for 1 min, increasing to 100% B over 7 min, and held for 2 min before returning to the start conditions. Injection volumes varied between 1 µL and 20 µL. The PRMs were detected by fluorescence using an excitation wavelength of 250 nm and an emission wavelength of 395 nm. Data were evaluated using ChemStation for LC Rev. B.04.01 SP1 from Agilent Technologies.

#### Qualitative analysis

The composition of PRM analogs in the samples was analyzed via ultra-high performance liquid chromatography coupled with high resolution mass spectrometry (UHPLC-HRMS). Here, a 1290 UHPLC system was coupled to a 6550 iFunnel Q-TOF LC/MS equipped with a dual Agilent Jet Stream (AJS) operated in the electrospray ionization mode (all Agilent Technologies, Waldbronn, DE). A Kinetex F5 column (100 × 2.1 mm, 2.6 µm, Agilent Technologies, Waldbronn, DE) was used at 30 °C with a flow rate of 0.4 mL/min. Eluent A was water, and eluent B was ACN:H_2_O (90:10, *v/v*) and both eluents contained 0.1% formic acid and 1 mM ammonium formate. The following gradient was applied: the starting conditions were 20% eluent B for 30 s, followed by a linear gradient reaching 67% B over 15.5 min. Thereafter the column was flushed for 3 min using 100% eluent B before returning to the starting conditions. Injection volumes ranged between 1 and 10 µL. The capillary voltage was set to 4000 V and the nozzle voltage to 500 V. The gas temperature was 130 °C and the drying gas flow was 14 L/min. The sheath gas temperature was set to 300 °C and a flow rate of 10 L/min. The nebulizer pressure was held at 2.07 bar (2.07*10^5^ Pa). The mass spectrometer was operated in full-scan positive ionization mode scanning *m/z* 50 to 1700 with 3 scans per second. Reference masses of *m/z* 121.05087 and *m/z* 922.00 were constantly infused into the ion source via a second nebulizer to ensure mass accuracy. The examined masses were adopted from Binzer et al. (2019). Data were acquired using MassHunter Workstation LC/MS Data Acquisition version B.06.01 and data evaluation was performed using Agilent MassHunter Qualitative Analysis B.10.00.

### Cell culture

Cytotoxicity assays were performed with the rainbow trout (*Oncorhynchus mykiss*) gill cell line RTgill-W1 obtained from Kristin Schirmer (Department of Environmental Toxicology, EAWAG, Dübendorf, CH). This cell line was cultured at 19 °C and sub-cultured every week, by first rinsing the flask with Versene (Thermo Fisher Scientific, Waltham, MA, US) and subsequent trypsinization using 0.25% trypsin/0.02% ethylenediaminetetraacetic acid in phosphate buffered saline (PAN Biotech, Aidenbach, Germany). The cells were centrifuged at 50 rcf and 21 °C for 3 min, the supernatant discarded, and the cells suspended in fresh medium prior to further usage. Two cultivation media were compared and tested, that differed both in their composition and the supplementation (supplementary information (SI) Table 1). The fully supplemented media are referred to as L-15 complete.

The human epithelial colon cell line HCEC-1CT was kindly provided by Jerry W. Shay, UT Southwestern Medical Center, Dallas, Texas, USA, and was used for cytotoxicity tests and live-cell imaging as previously described (Del Favero et al. 2018; Rebhahn et al. 2022). The cells were cultivated in Dulbecco’s Modified Eagle’s Medium (DMEM (Thermo Fisher Scientific, Waltham, USA)), supplemented with essential nutrients and growth media, at 37 °C and 5% CO_2_. For 500 mL DMEM 10 mL Medium 199 (10x), 10 mL HEPES buffer solution 1 M, 5.2 mL Insulin-Transferrin-Selenium-G Supplement (Thermo Fisher Scientific, Waltham, USA), 10 mL HyClone™ Cosmic Calf™ Serum (GE Healthcare Life Sciences HyClone Laboratories, South Logan, USA), 0.6 mL gentamycin solution (Sigma Aldrich GmbH, St. Louis, USA), 100 µL recombinant human epidermal growth factor (100 µg/mL, Szabo-Scandic HandelsgmbH & Co KG, Vienna, Austria) and 100 µL hydrocortisone (5 mg/mL, Merck KGaA, Darmstadt, Germany) were added. Cells were passaged every 2–3 days when a confluence of 80% was reached. HCEC-1CT cells were first rinsed with phosphate buffered saline and then detached with Accutase^®^ (Corning, Manassas, USA).

Both cell lines were cultured in cell culture flasks using the “cell + surface” (Sarstedt AG & Co KG, Nürnbrecht, Germany) of various sizes (T-25 to T-175). Cytotoxicity assays were performed in 96-well polystyrene cell culture plates (cell + surface, flat base, order number 83.3924.300; Sarstedt AG & Co. KG, Nürnbrecht, Germany), whereas live-cell-imaging assays were performed with 96-well flat clear bottom black polystyrene TC-treated microplates from Corning^®^ (order number: 3603, Corning, USA).

#### Cytotoxicity testing

RTgill-W1 cells were seeded at a density of 2*10^4^ cells/well and HCEC-1CT cells at 5*10^3^ cells/well in 96-well plates and grown for 48 h. PRM samples in EtOH were diluted 1:200 in culture medium to achieve a final EtOH concentration of 0.5%. Since PRM are light labile exposure to sun light was avoided and the exposure time to artificial light were kept to a minimum. Cells were exposed to 100 µL of different concentrations thereof, for 3 or 24 h in the dark. The CellTiter-Blue^®^ (CTB) assay was performed to measure the metabolic activity of the cells, according to the manufacturer’s instructions. The CTB dye was diluted 1:10 (v/v) in cell culture medium and applied to the cells for 1 h in the dark.

Where applicable, prior to starting the CTB assay, the lytic potential of PRMs was analyzed with the lactate dehydrogenase (LDH) assay (Thermo Fisher Scientific, Waltham, USA). The measurements were performed according to the manufacturer’s protocol.

Additionally, cell protein content was assessed for 24 h incubations, using the sulforhodamine B (SRB) assay. In short, the cells were rinsed with Dulbecco’s Phosphate Buffered Saline (Thermo Fisher Scientific, Waltham USA)), fixed with trichloroacetic acid and incubated for 1 h at 4 °C. The plate was then washed four times with distilled water and dried overnight. The fixed cells were stained with SRB reagent for 1 h at room temperature, after which it was discarded, and the plate was rinsed with water followed by 1% acetic acid in water. The plate was again dried overnight, and finally, Tris base was used to dissolve the stained proteins. After shaking the plate for 5 min, the absorbance was taken at 570 nm.

For PRM toxicity in ion-free media, crystal violet (CV) dye was used instead of CTB. Cells were seeded and exposed to test solutions as described before. After incubation, wells were aspirated, and the cells fixed with ice-cold EtOH for 10 min at 4 °C. This was discarded, and 0.1%-CV solution (in EtOH) was used to stain the cells for 5 min. Subsequently cells were rinsed with water and dried overnight. De-staining solution (1% acetic acid in EtOH) was added to each well and absorbance was measured at 595 nm.

#### Live-cell imaging

The cell nuclei and membranes were visualized using Hoechst 33,258 and Cell Mask™ Deep Red plasma membrane stain (Fisher Chemical, Loughborough, UK), diluted 1000-fold in Live Cell Imaging Solution (LCIS; Thermo Fisher Scientific, Waltham, USA). After staining for 30 min at room temperature (RTgill-W1) or at 37 °C and 5% CO_2_ (HCEC-1CT), cells were rinsed first with the respective media, and images were taken right before adding the substances (*t*_0_), and again 1.5 h (*t*_1_) and 3 h (*t*_2_) after starting the exposure to PRMs. Sample dilutions were prepared in LCIS, normal external solution (NES), and the ion-free media. Images were acquired using a BioTek Lionheart FX (Agilent, Santa Clara, CA, United States) automated microscope. Data evaluation was performed with ImageJ Software and BioTek Gen5 Software for imaging and microscopy.

#### Ion-free media

Specific ions (Na^+^, Ca^2+^ or Cl^−^) were chosen to be omitted entirely for cytotoxicity testing and fluorescence microscopy, adapting protocols previously described (Del Favero et al. 2012). The composition of each medium can be found in SI Table 2. Additionally, LDH and CV assays in HCEC-1CT were performed for different concentrations of B-type PRM in the selected ion-free media, as well as NES, LCIS, and HCEC-1CT culture medium.

### Statistics

CTB and LDH results were tested for normality via the Shapiro–Wilk Test, significance (* = *p* < 0.05; ** = *p* < 0.01; *** = *p* < 0.001) was calculated with One Way ANOVA, followed by the post hoc Fisher´s least significant difference test using OriginLab.

## Results

### Chemical analysis

The PRM content was analyzed semi-quantitatively, and qualitatively. The mycotoxins fumonisin B_1_ and B_2_ were used as external calibration standards due to the lack of PRM standards, as these also contain a primary amine, for which individual calibration curves were created. A-type PRM eluded at a retention time of 7.6 min, B-type between 6.75 and 7.65, and C-type PRMs had a retention time between 6.1 and 7.4 min. PRM analogs could not be distinguished in this method. Peaks with an area larger than 50 were excluded, and the samples were diluted for repeated measurements. From the external calibration, approximate concentrations of the ichthyotoxins could be calculated, and those are provided in SI Table 3. SI Table 4 states the PRM profiles that were determined using UHPLC-HRMS analysis. This was also performed to estimate the purity of the solutions. The most common ion species observed were the double charged ions [M + 2H]^+2^, [M + NH_4_ + H]^+2^ and [M + Na + H]^+2^. In case of B-type PRMs also, the single charged [M + H]^+^ was observed. Beside the exact masses, also the quite distinctive isotopic patterns were taken into consideration to confirm the presence of PRMs. The isotope distribution of PRMs is very specific due to the high number of C-atoms and the occurrence of Cl-atoms. For one of the two UTEX-2797 extracts, A2, no more than four analogs could be identified, with the majority, 81%, attributed to the A-type prymnesin with 3 Cl-atoms incorporated and one pentose moiety (short form: PRM-A (3 Cl) + pentose). For extract A1, on the other hand, ten analogs were identified. The highest concentration was 41% for PRM-A (3 Cl) + pentose. We currently have no explanation for the difference in the PRM-pattern of the same strain which was cultivated years apart. Sample A3, from unknown strain origin (Sigma Aldrich), was found to consist of seven different analogs, with PRM-A (3 Cl) + 2 pentose + hexose being the most abundant, at 63%. The PRM profiles for the B-type extracts of K-0081 consisted of eight different analogs. Approximately half of the PRM content, though, was comprised of PRM-B (1 Cl) + hexose. Generally, PRM compositions matched perfectly between extraction replicates of each B-type *P. parvum* strain. Only for sample B4, which was harvested from K-0081 3 years prior to the other B-type extracts, differed slightly from its counterparts. Lastly, sample C1 of the strain RCC-1436 was found to contain at least seven analogs, while for C2 from strain RCC-191, more than ten analogs of C-type PRMs could be identified. Interestingly, PRM-C (4 Cl + DB) + pentose appeared to be the most abundant in both cases.

In order to identify whether potencies differ between PRM analogs, purified solutions were also analyzed and subsequently tested for cytotoxicity. Both, the A-type single substance solution from the UTEX-2797 strain, sA1, and a purified B-type sample harvested from strain K-0081, sB1, consisted of only two analogs. One of which was significantly more dominant than the other, for both samples, respectively. For sB2, three analogs could be identified, where PRM-B (1 Cl) + 2 hexose made up for 79%. An overview of the identified PRM analogs is provided in SI Table 4.

### Cytotoxicity

The cytotoxic potential of *P. parvum* extracts and PRM single compounds was tested in a CTB cell viability assay, for which the optimal incubation time of 60 min was determined. A final EtOH concentration of 0.5% was applied as solvent control, which was the point of reference for SRB and CTB calculations. For LDH analyses, a maximum LDH release (maximum lysis) of the cells was determined, which was ultimately used as reference for potency analyses. Triton™ X-100 (short Triton X, Sigma-Aldrich, St. Louis, MO, USA) served as positive control. For CTB assays, 0.05% (v/v) in medium were applied to the RTgill-W1 cells and 0.1% (v/v) to HCEC-1CT, and in LDH assays, 0.1% (v/v) Triton X was used. The setup for the 24 h incubation was the same as for 3 h, only Triton X concentrations were changed to 0.025% in RTgill-W1 cells, and 0.075% (v/v) for HCEC-1CT cells.

#### RTgill-W1

The fish gill cell line RTgill-W1 was exposed to A-, B, and C-type PRM samples and also the impact of two culture media compositions (recipe 1 and recipe 2) was assessed. Toxin potencies did not differ significantly in the two media (SI Fig. 1). Incubation times of 3 h and 24 h were compared, and the effective concentration 50 (EC_50_) values differed only by a factor of around 2.2 (SI Table 5). Given the small differences, a 3 h incubation was chosen for the remaining experiments.

**Fig. 2:**
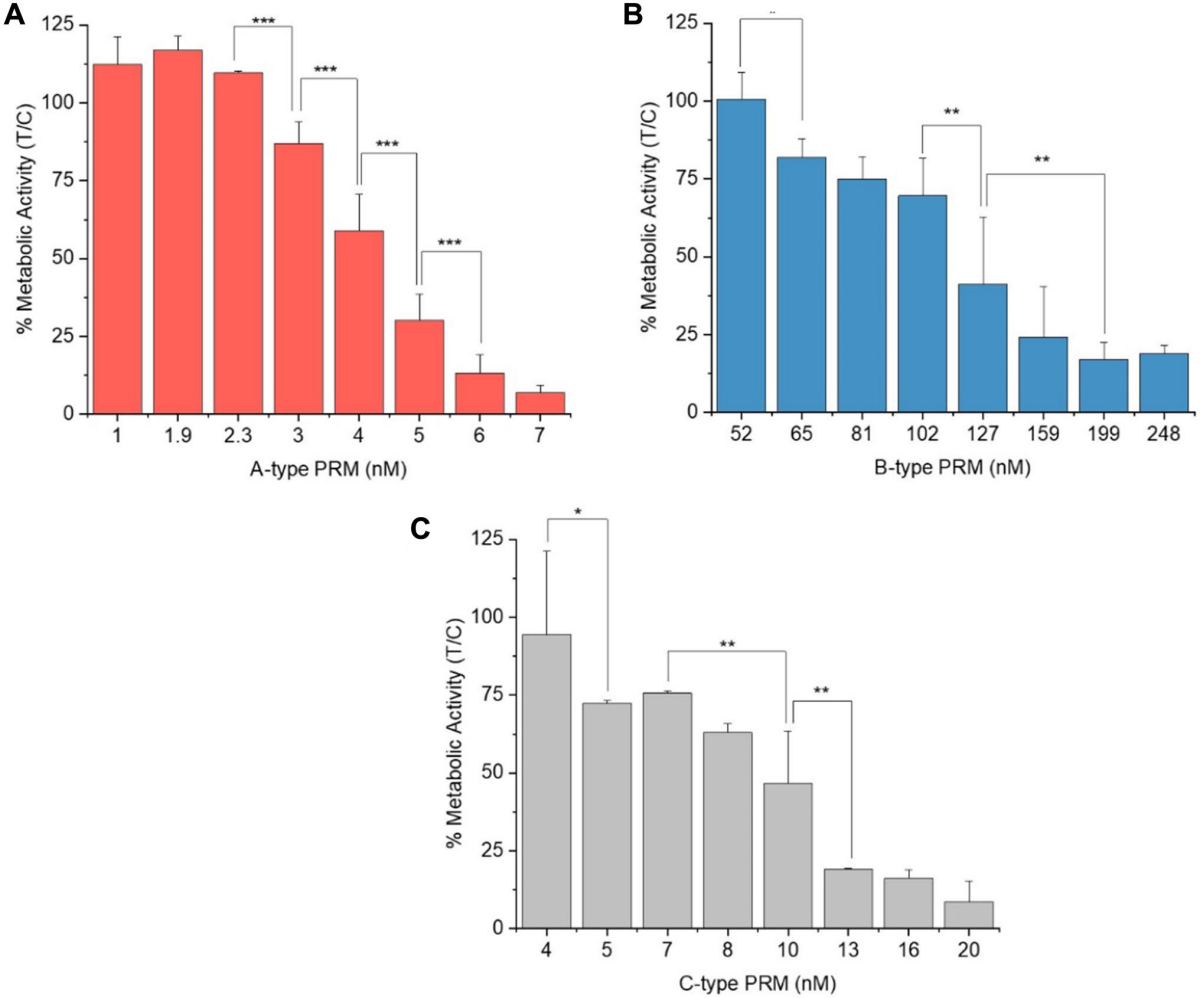
RTgill-W1 cell viability after 3-h exposure to prymnesins (PRM). **A** shows data of the A-type solution A3 from an unknown strain, **B** results for the K-0081 extract B3, and **C** the RCC-191 extract C2. Data are represented as mean ± SD, n ≥ 3

**Fig. 3:**
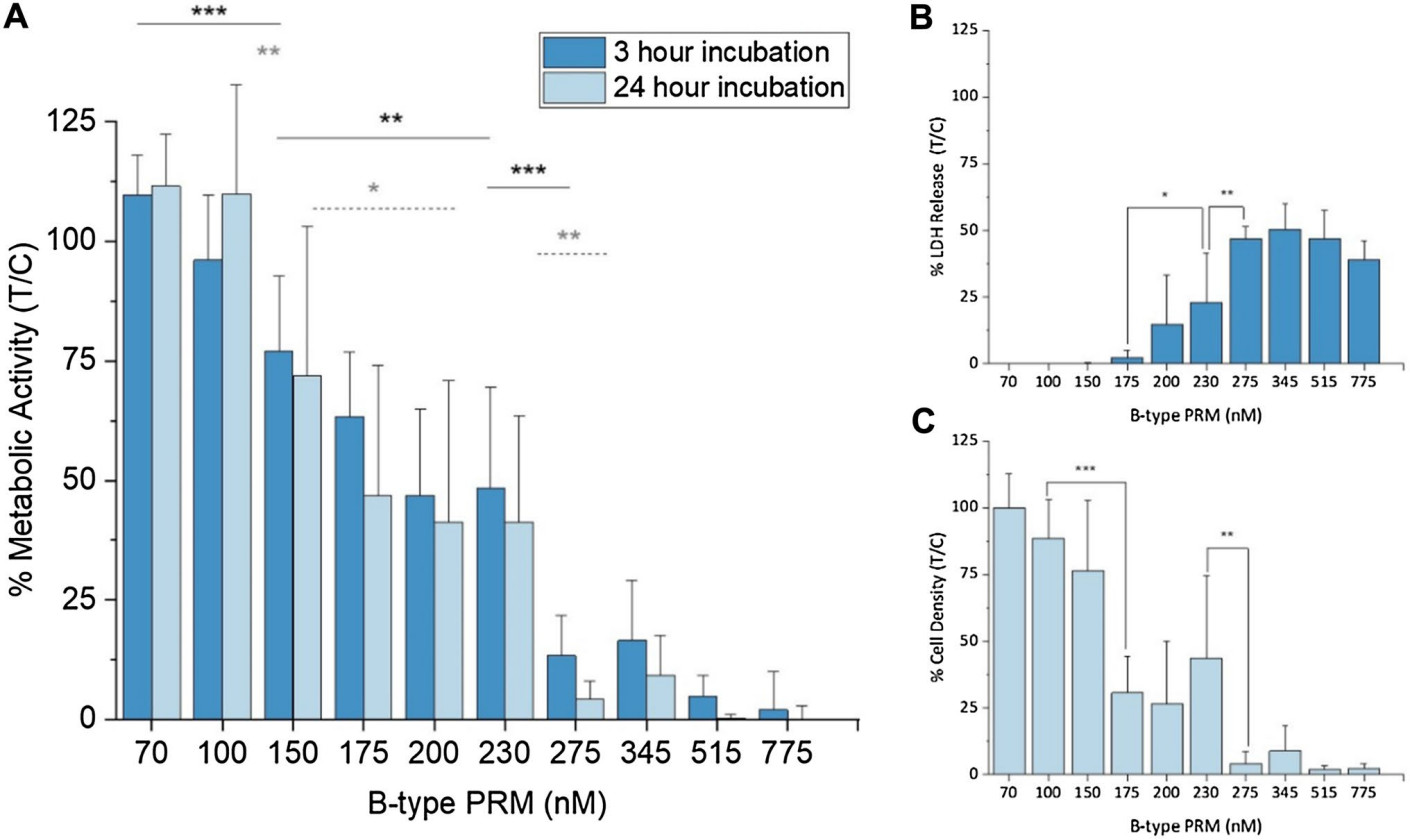
Comparison between HCEC-1CT cell viability after incubation with B-type prymnesin (PRM) extract B1 obtained from *Prymnesium parvum* strain K-0081 for 3 h and 24 h (significances displayed with straight and dotted lines) (**A**). **B** shows the cell lysis corresponding *to* a 3-h incubation measured in the lactate dehydrogenase (LDH) assay, and **C** the protein/cell density after 24-h exposure to PRMs, using the sulforhodamine B assay. Data are represented as mean ± SD of n ≥ 3

Cells were exposed to the A-type PRM solution from Sigma Aldrich (A3), B-type extract of the K-0081 strain (B3), and a C-type PRM extract of RCC-191 (C2) diluted in medium following recipe 2. A cytotoxic effect could be measured for all the tested samples, some of which are shown in Fig. 2. The A-type sample, A3, showed a clear increase in cytotoxicity with increasing concentrations (Fig. 3A). Yet, at the lowest concentrations an elevation of RTgill-W1 metabolic activity was observed. Cell viability was decreased to about 50% at a concentration of 127 nM of the B-type PRM extract B3. Extract C2, from the C-type producing strain RCC-191, already reduced cell viability to 75% at 5 nM, and had an EC_50_ of 9.8 ± 0.8 nM. The effects of PRMs in the fish gill cell line were compared to the human-derived HCEC-1CT cells (SI Table 3) and RTgill-W1 cells were about twofold more sensitive to the ichthyotoxins than the human cell line.

#### HCEC-1CT

*P. parvum* extracts were tested on non-tumorigenic human colon epithelial cells. HCEC-1CT cells were exposed to the B-type extract B1 of *P. parvum* strain K-0081 (Fig. 3). CTB and LDH assays were performed for 3 h incubations, and CTB and SRB for 24 h incubations (Fig. 3A). CTB measurements after 3 h exposure showed very low viability for the highest concentrations, while the lowest seemingly did not alter cell viability. An EC_50_ of 170 ± 9 nM was calculated with RStudio (packages “drc”, “ggplot2”). For the 24 h incubation, the EC_50_ was calculated to be around 145 ± 7 nM. These findings were mirrored in the corresponding LDH data obtained for the 3 h incubations (Fig. 3B), and SRB results (Fig. 3C) for the 24 h incubation. Based on the cytotoxicity results obtained for B-type PRMs, all following exposures were limited to 3 h. Extract A1 from strain UTEX-2797 was applied in concentrations ranging from 5 to 40 nM (SI Fig. 2A), and a strong cytotoxic potential could be observed. An EC_50_ of 12.7 ± 0.3 nM was calculated. The C-type PRM extract of the *P. parvum* strain RCC-191 was also tested for their cytotoxic potential towards HCEC-1CT cells (SI Fig. 2B). A continuous decrease in cell viability could be observed, although 100% cell death was not reached.

**Fig. 4:**
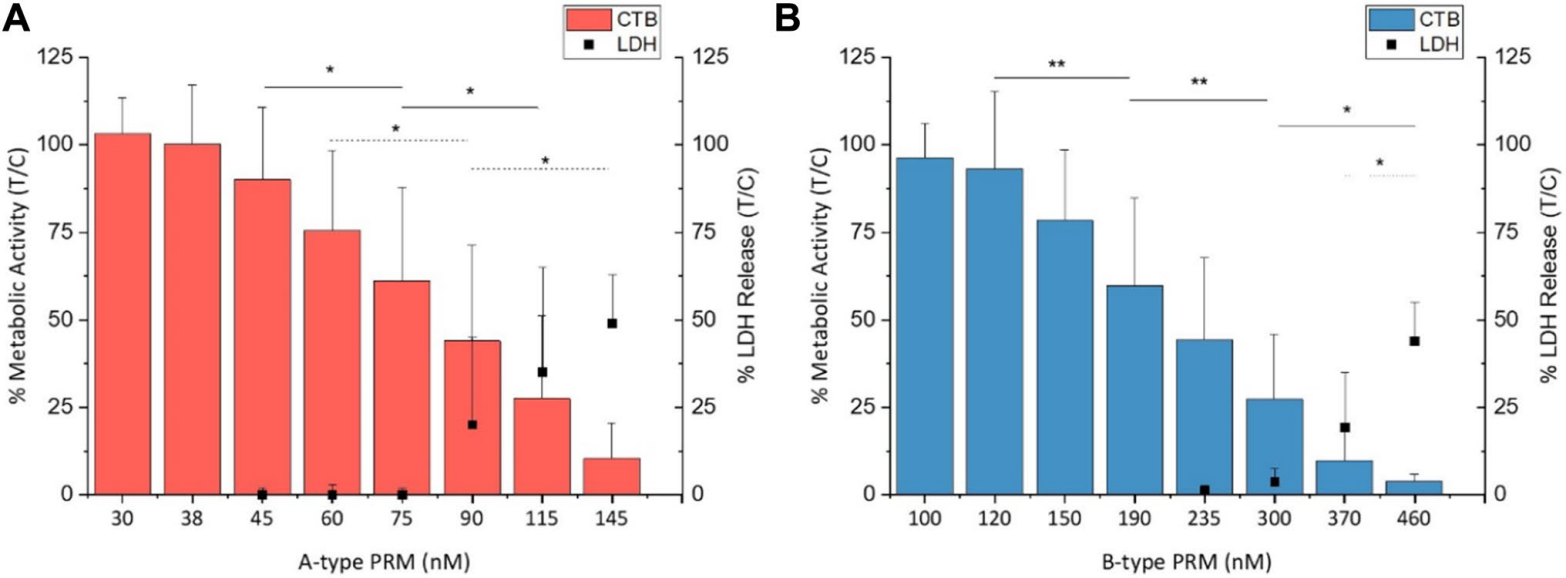
Cell viability and membrane integrity of HCEC-1CT cells after 3-h incubation with single compounds of A-type prymnesin (PRM) sA1 derived from UTEX-2784 (**A**). In **B** the same can be seen for B-type PRM single substance solution of sB1. Cell titer blue (CTB) (straight line) and lactate dehydrogenase (LDH) (dotted line) assay data are represented as mean ± SD of n = 5

The single compound samples, two B-type (sB1 and sB2) and one A-type (sA1), were also assessed for their effects on cell viability (Fig. 4). An EC_50_ value was estimated at 76 ± 34 nM for sA1 (Fig. 4A). Exposure to sB1 and sB2 also resulted in a continuous decline of cell viability, an EC_50_ values of 220 ± 30 nM and 270 ± 160 nM were calculated, respectively. In the LDH assay, however, cell damage could not be detected until a viability of ≤ 45% was attained in the CTB. For better comparison, the EC_50_ values obtained from CTB assays were translated into the sum of PRM analogs in µg/L (SI Table 3 and SI Formula 1).

Finally, a comparative assessment was performed between *P. parvum* extracts obtained at different dates. This was performed to measure possible variabilities between extractions, as well as influences of storage time on toxicity (SI Fig. 3). Both extracts of the UTEX-2797 strain, harvested 3 years apart, showed a distinct positive correlation between PRM concentration and cytotoxic effects. Nonetheless, extract A1 was significantly more potent than A2; 16 nM of A1 reduced cell viability to 15%, whereas exposure to the same concentration of A2 only achieved a decrease to 73%. Interestingly, this considerable difference in potency could no longer be observed for the highest concentration. It was striking that the prymnesin profile differed between these two strains as previously mentioned (SI Table 4). Similarly, four B-type PRM extracts derived from strain K-0081 were tested for biological variance (Fig. 3B). Unlike the results for the A-type extracts, the responses of HCEC-1CT cell exposure to the B-type samples were nearly identical and also the prymnesin profile was quite similar (SI Table 4).

### Cytotoxicity in ion-free media—fluorescence microscopy

RTgill-W1 cells and HCEC-1CT cells were stained with Hoechst and CellMask™ for live-cell imaging to visualize the cell nuclei and the cell membrane. A concentration of 6 nM of A-type sample A3 was applied to RTgill-W1, and three concentrations of B-type PRM extract B1 were applied to the HCEC-1CT cells. Toxin solutions were prepared in LCIS (only for HCEC-1CT), NES, Na^+^-free, Cl^−^-free, and Ca^2+^-free medium.

**Fig. 5:**
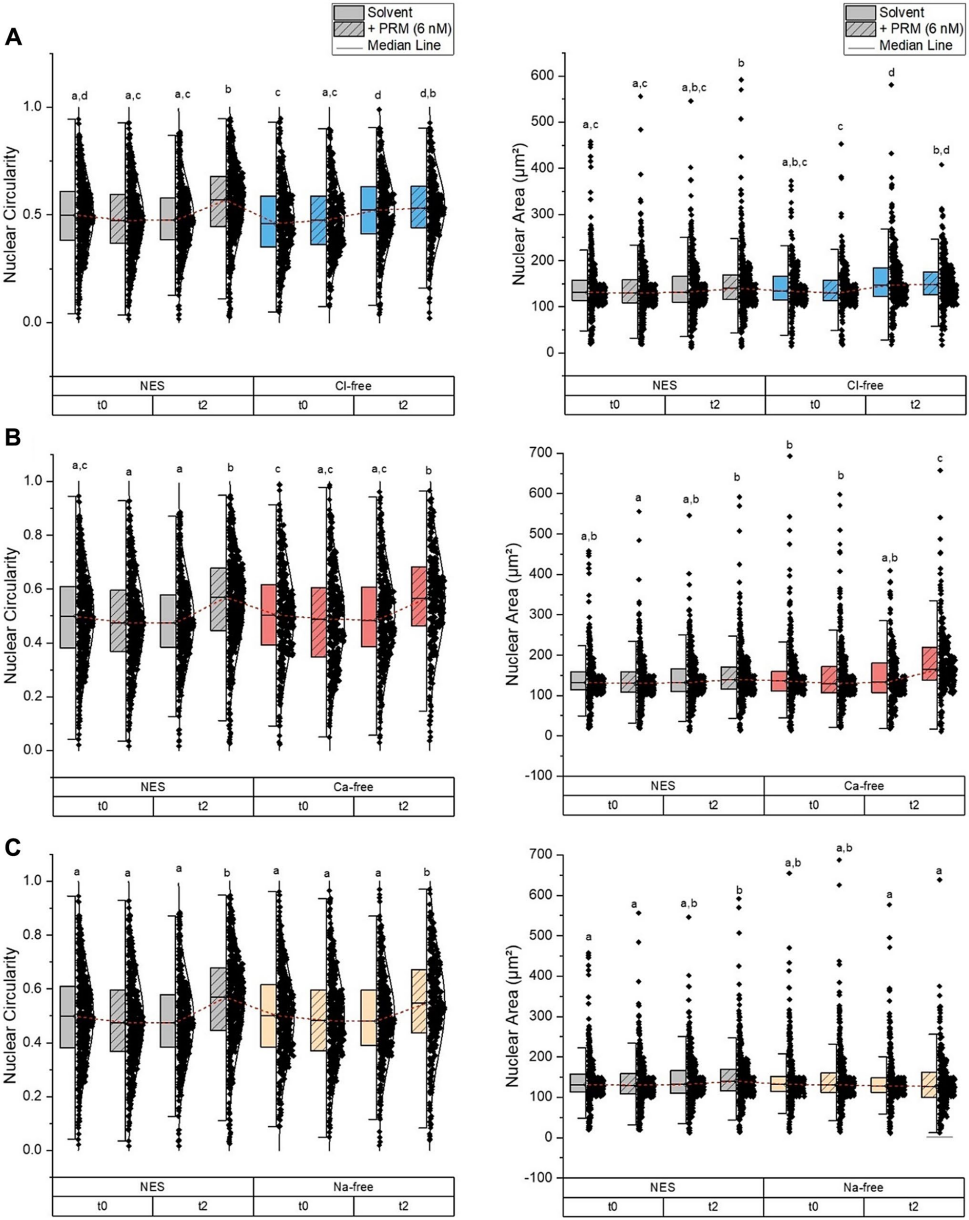
Nuclear circularity and nuclear area of RTgill-W1 cells after 3-h exposure to A-type prymnesins (PRMs) of solution A3 in normal external solution (NES), Cl^−^-free medium (**A**), Ca^2+^-free medium (**B**), and Na^+^-free medium (**C**). Graphs show all data points collected from biological replicates of n ≥ 3

Image analyses for RTgill-W1 cells were performed with the aid of Gen5 Software as well as the ImageJ Software. The time point t_2_ (3 h) was chosen for analysis of the effects of the treatment on the RTgill-W1 cells. The nuclear morphometric parameters like area and circularity were evaluated as indicators of the osmotic stress (Finan and Guilak 2009) for each cell that was counted within one image using Gen5. All data points for these two parameters, gathered throughout several biological replicates, were plotted for each medium (Fig. 5). Additionally, the ratio between cellular and nuclear area was assessed, by random manual selection of twelve cells per image using ImageJ (Fig. 5). Exposure of RTgill-W1 cells to PRMs lead to an increase in nuclear circularity in the control medium (NES). This effect was just as pronounced in Ca^2+^-free and Na^+^-free medium (Fig. 5). The impact on nuclear circularity of RTgill-W1 cells exposed to PRMs in Cl^−^-free medium could also be observed, albeit not as evident. Although Cl^−^-free medium in general caused a slight increase in circularity, as can be seen in the solvent control in Fig. 5A, which was not observed for the other media. Example images for fluorescent staining are provided in SI Fig. 4 and phase contrast images are shown in SI Fig. 5.

Interestingly, incubation of RTgill-W1 cells in Cl^−^-free medium also resulted in a slightly larger nuclear area, compared to in NES medium. This could indicate that the change in nuclear morphology observed in medium lacking Cl^−^ ions may be due to the medium itself, as opposed to the exposure to PRMs. It is worth mentioning that PRM-exposure in Ca^2+^-free medium had an effect on the nuclear area as well (Fig. 5). Here, however, the change in area was not observed in the solvent control. The ratio of the cell area versus the nucleus area is provided in SI Fig. 6.

For analysis of the HCEC-1CT cells, the total cellular area per well was divided by the number of nuclei detected to obtain the average area per cell. This value was used for comparison between the different testing conditions and time points. Each timepoint was related to LCIS without PRMs at t_0_ (before incubation). Images were taken after 1.5 h (t_1_) and 3 h (t_2_). At t_1_, the mean cellular area decreased after exposure to PRMs in all media but the Cl^−^-free (SI Table 6). A further decline was observed when the incubation time was extended for another 1.5 h (total of 3 h). For PRMs in the Cl^−^-free medium, a slight increase in area of about 14% was observed. SI Figs. 7, 8, 9, 10, 11 provide representative images of the solvent control in the different media at *t*_0_ and *t*_3_ and the two higher PRM-concentrations tested (*t*_3_).

Building on the assumption that Cl^−^ could be relevant for PRM toxicity, the impact of the lack thereof was assessed in cell viability analyses for HCEC-1CT cells, as the effect was more pronounced in this cell line. Since the CTB dye solution contains NaCl, alternative cell viability assays were required, and it was found out that neither the LDH- nor the CV-assay interfere with the cellular osmotic equilibrium. The B-type PRM extract was prepared in HCEC-1CT culture medium, LCIS, and Cl^−^-free medium (SI Table 7). Three PRM concentrations were tested per condition. The LDH assay showed a clear lytic effect for the highest concentration of PRMs in culture medium and in LCIS, albeit to a much lower extent. No significant toxicity was measured for the Cl^−^-free medium, which is in accordance with the live imaging data. The influence of ions was also tested for A-type PRMs. The solution A3 of unknown strain origin was tested for its toxicity in NES, Na^+^-free, Ca^2+^-free, and Cl^−^-free medium. As expected, a high cytotoxic potential was measured for all media except for the medium containing no Cl^−^. Cellular changes upon exposure were captured with bright field images (SI Fig. 12).

## Discussion

All prymnesin extracts of *P. parvum* tested in this project were cytotoxic. Only minimal differences in the potency were observed between the two exposure times (3 h and 24 h), indicating that PRMs act quickly. For instance, the EC_50_ values of the K-0081 extract B1 only differed by 25 nM between the two incubation times in HCEC-1CT (170 ± 9 nM and 145 ± 7 nM for 3 and 24 h, respectively) and for 68 nM in RTgill-W1 (see SI Table 5). This similarity indicates that even a short-term interaction with PRMs can cause substantial adverse effects, and that longer exposure would not significantly increase the effect in vitro. This theory was highlighted during fluorescence live-cell imaging, where cells visibly changed their morphology after a 1.5-h exposure to PRMs already. These findings are supported by the observations made by Svendsen et al. (2018), who found that short-term exposure to *P. parvum* microalgae had considerable negative effects on rainbow trout fish, which were not reversible.

Generally, the fish gill cell line was more susceptible to the PRMs than HCEC-1CT cells, albeit to a small degree. This observation also withstood when compared to the data described by Rasmussen et al. (2016a), where RTgill-W1 cells were exposed to A- and B-type PRMs for three hours. Either way, the experiments here showed that *P. parvum* toxins can be just as effective towards human-derived cells. This should be kept in mind for future HAB episodes although no human incident has been reported to this day. In the *P. parvum* bloom in the Oder River in August 2022, beside 360 t of dead fish, also the death of freshwater bivalves, other mollusks as well as birds, ducks, beavers and other wildlife was reported, but could not be directly linked to the HAB (Free et al. 2023). Nonetheless, algal toxins often accumulate in bivalves and shellfish (not shown for PRMs yet), which are then consumed by humans (Hallegraeff 1993; Rasmussen et al. 2016b). Thus, it is not entirely inconceivable that humans can be exposed to toxic PRMs as well (Manning & La Claire 2010; Vasas et al. 2012). Should humans consume contaminated shellfish, PRMs may pass through the gastro-intestinal tract, where the pH is naturally low; pH 1.3–1.7, in the stomach (Dressman et al. 1990; Russel et al. 1993). How PRMs would behave in this environment is debatable. Higher hemolytic potential has been described for PRMs at lower pH, yet maximum ichthyotoxicity was observed at pH 9 (Igarashi et al. 1998; Manning & la Claire 2010; Ulitzur and Shilo 1964). Another possibility for humans to get in touch with PRMs is through bathing in contaminated water. Thus, assessing an impact of PRMs on skin/skin cells would be the next step towards understanding their hazard to humans.

A clear order of potency was observed in both HCEC-1CT and RTgill-W1 cells, with A-type PRMs being the most potent, and B-type the least. These findings are in accordance with the previously published results by Rasmussen et al. (2016a). For A-type PRM samples, concentrations were usually in the low nmol/L region, and C-type PRM concentrations were in a similar range. Meanwhile to achieve an equivalent cytotoxic effect, B-type PRM had to be applied in much larger molarities. Identifying the *P. parvum* strain is thus a crucial detail for assessing the risk of a *P. parvum* bloom. In yet another study, acute ichthyotoxicity of five strains of *P. parvum* in rainbow trout fish was analyzed, in which strains UTEX-2797 and K-0081 were found to be the two most potent (Blossom et al. 2014). This unexpected outcome, with a B-type strain being among the most toxic, is contrary to what has been known about PRM toxicity so far. This led to further investigations, and the studies showed that the cellular content of PRMs differs substantially among the *P. parvum* strains (Binzer et al. 2019; Svenssen et al. 2019). As an example for B-type PRM, cells of the strain K-0081 could contain up to 30 times more toxin than K-0374 (Medić et al. 2022; Svenssen et al. 2019).

A number of environmental factors such as pH, irradiance/sunlight, temperature, salinity, and nutrient availability have an impact on the growth of *P*. *parvum*, toxin content and toxin production (Hill et al. 2020; Manning and La Claire 2010; Medić et al. 2022; Svenssen et al. 2019; Taylor et al. 2021). However, many more studies are required to understand the production and release of PRMs. For instance, Medić et al. (2022) observed that the production of PRMs was directly coupled to the growth rate in the strain K-0081 grown at different irradiances, while this was not the case of the K-0734 strain (Medić et al. 2022).

Little is understood of the biosynthesis of marine secondary metabolites, yet, given their polyketide structure, the involvement of polyketide synthases is highly plausible (Anestis et al. 2021; Manning and La Claire 2010). The analyses of Anestis et al. (2021), showed a large number of polyketide synthase genes in nine *P. parvum* strains, although no clear connection to cellular PRM content could be found. Nonetheless, it was implied that toxin production in *P. parvum* comes at a metabolic cost (Anestis et al. 2021). Environmental conditions not only affect PRM synthesis and release, but also its stability, as shown for culture filtrates that lost acute toxicity towards fish after exposure to sunlight (Blossom et al. 2014; Taylor et al. 2021). Regarding these environmental factors, uniformity is needed for microalgal cell culture conditions, as they can significantly affect the toxin production itself and consequently toxicological investigation (Brooks et al. 2010; Hill et al. 2020; Manning and La Claire 2010).

Considering the above, toxin quantification prior to toxicological research is just as crucial. One challenge for quantification, however, is posed by the extraction process, among other reasons due to low natural production and amphipathic properties of the compounds (Andersen et al. 2017; Svenssen et al. 2019; Tillmann 2003). A lack of reliable and commercially available standards combined with missing standardized isolation methods are the limiting factors for proper quantification of ichthyotoxins (Brooks et al. 2010; Manning et al. 2013; Svenssen et al. 2019). The method applied in this project was described by Svenssen et al. (2019), who reported an apparent recovery of 58%, which highlights the need for further improvement of ichthyotoxin extraction from biomass (Andersen et al. 2017; Svenssen et al. 2019). Loss during sample preparation can even hinder the applicability of those extracts in cytotoxicity assays, as the obtained concentrations might be too low, particularly when natural toxin production is already low (Svenssen et al. 2019). Taking this information into account, all given concentrations throughout this study should be interpreted as estimates. It is thus difficult to directly translate the results from the assays performed here into their potency in vivo. What can be done, though, is to draw relations between the strains and the PRM analogs produced, i.e. fish exposed to the RCC-191 strain could be expected to suffer less than when exposed to RCC-1436 under the condition that PRMs are produced in the same quantities.

When different strains producing the same type of PRM were compared, the identified PRM profiles differed greatly. This variability was mirrored in the cytotoxic potential. Extracting PRMs from the same microalgal biomass at different times in case of strain K-0081 had effectively no impact on the PRM profile and consequently the toxic properties (SI Table 4 and SI Fig. 3B). Although, when the relative amount of the same analog between samples differed in 10% or more (e.g., extract A1 vs. A2, SI Table 4), significant changes in toxicity were observed (SI Fig. 3A). Apparently, the kind of analogs produced by *P. parvum* are essential when it comes to their cytotoxic potential. Whether this toxicity depends on a particular type of analog and its relative quantity or a specific combination of several analogs could not be discerned. The most toxic sample used in this study was the A-type solution from unknown strain origin, with only 4.0 ± 0.2 nM PRMs to achieve a 50% effect. The second most potent A-type sample was a UTEX-2797 extract (A1). What both of these samples had in common, was the relatively high amount of PRM-A (3 Cl) + 2 pentose + hexose, with 23 and 63% for the extract and the single compound solution respectively. The second most abundant analog in both samples was PRM-A (3 Cl) + pentose. Whether it is a combination of these two that influences potency or the analogs themselves that are more cytotoxic than others remain unclear. By looking at the PRM profiles of the tested samples, cytotoxicity was not related to the sheer number of analogs produced. Which could be seen in the C-type extracts from RCC-1436 and RCC-191. Seven kinds of analogs were identified for the former, and fourteen for the latter, yet the higher number did not cause higher cytotoxic effects. Likewise, all single compound solutions consisted of fewer PRM analogs than the *P. parvum* extracts, yet in general, extracts were more potent. This phenomenon may not be explained by the number of analogs, but by the presence of other potentially toxic undetected compounds in the extracts. It can be assumed that *P. parvum* extracts contain molecules that are not identified as PRMs, which may or may not enhance the toxic potential of PRMs. Nevertheless, assessing the single compound solutions showed that even purified PRMs are cytotoxic, and thus the harmful effects observed during HAB events can be, at least in part, attributed to the presence of these ichthyotoxins. Further cytotoxicity evaluations of PRMs should focus on the individual analogs, in order to obtain information about the role they play in overall toxicity. Ideally, single analogs can be tested, followed by combinatorial assessments. With these results, it can be interpreted that the harmfulness of *P. parvum* strains is connected to (1) the type of PRM (A, B, or C), (2) the PRM profile and (3) PRM abundance (for HAB episodes).

The impact of PRM extracts and single compound solutions could be observed via live-cell imaging experiments, with visible effects on their morphology, probably related to their viability. As already established previously, PRMs cause pore formation in the cell membrane, through a yet unknown mechanism, which increases their permeability (Ulitzur and Shilo 1966; Yariv and Hestrin 1961). Higher ichthyotoxicity of PRMs in the presence of Ca^2+^ and Mg^2+^ had been observed previously, and it was postulated that these cations act as cofactors or activators for these ichthyotoxins (Shilo 1981; Ulitzur and Shilo 1964; Yariv and Hestrin 1961). In RTgill-W1 cells, however, the absence of Ca^2+^ seemed to exacerbate the effects caused by PRMs. As this was a cell culture experiment, it should be noted that Ca^2+^ plays a crucial role in cell–cell adhesion as well as adhesion to the plate surface (Takeichi and Okada 1972; Weiss 1960). Removing Ca^2+^ from the medium could therefore impact the attachment of RTgill-W1 cells to the well bottom. The effect observed for PRMs in Ca^2+^-free medium may thus be at least partially attributed to this fact. Considering that no significant difference for the nuclear parameters was measured between the Ca^2+^-free solvent control and the control medium NES, it can be assumed that the condition “PRM-Ca^2+^” was indeed responsible for the observed outcome. It appears that HCEC-1CT cells were affected differently by PRMs than RTgill-W1 cells, not only because of differences on a structural level, but potentially because of differences in regulatory processes of these cell lines. For HCEC-1CT cells, the absence of Na^+^ or Ca^2+^ had no impact on PRM cytotoxicity. Removing Cl^−^ from the medium, however, decreased cytotoxicity in HCEC-1CT cells. As for the RTgill-W1 cells, the changes in the nuclear parameters upon incubation in Cl^−^-free medium were possibly caused by the medium itself, as the same changes could be observed in the Cl^−^-free solvent control. During preparation of the most promising recipe for the Cl^−^-free medium, it could be observed that the viability of RTgill-W1 cells is strongly affected by the absence of Cl^−^ ions, making it difficult to assess the impact of PRMs in a medium lacking this essential ion. Evidently, PRM toxicity is achieved through creating an osmotic imbalance after damaging the plasma membrane (Ulitzur and Shilo 1966). One explanation could be that PRMs interfere with Cl^−^ channels, which can regulate cell volume, control the ionic composition of cell plasma, or cellular pH (Jentsch et al. 2002; Verkman and Galietta 2009). Given that the presence of Ca^2+^ ions increased the toxicity in fish and their absence in this in vitro assay strengthened the toxic effects, it can also be suggested that PRM toxicity is intertwined with the Ca^2+^ regulation of gill cells (Shilo 1981; Ulitzur and Shilo 1964; Yariv and Hestrin 1961).

Fish are particularly dependent on osmoregulation and have evolved highly efficient ionoregulation systems in which gills are a key figure (Hwang et al. 2011). The osmoregulatory systems in fish are highly intricate and surpass the scope of this paper. To give a brief description though, fish gill cells can roughly be divided into two major cell types, ionocytes and pavement cells (Flik et al. 1997; Goss et al. 1995; Hwang et al. 2011). Active ion transport happens mainly in ionocytes, where Na^+^ and Cl^−^ uptake is regulated via the Na^+^/K^+^-ATPase (Lin et al. 1994; Marshall 1995, 2002). The vacuolar-type H^+^-ATPase, supports the transport of ions through maintaining the cellular pH, generating electrochemical gradients, and indirectly contributing to ion exchangers (Hwang and Lee 2007; Hwang et al. 2011; Marshall 2002). This H^+^-ATPase is activated by external Ca^2+^ and can be downregulated by high external Na^+^ content (Lin et al. 1994). Ca^2+^ uptake involves a Ca^2+^ channel and is linked to Na^+^ transport (Flik et al. 1997; Hwang et al. 2011; Marshall 2002). Gills are the main site for Ca^2+^ uptake in freshwater fish, and Ca^2+^ channels have been found in both pavement cells as well as ionocytes (Hwang et al. 2011; Shahsavarani et al. 2006).

Importantly, RTgill-W1 cells lack the characteristics of pavement cells and ionocytes, for they are apparently derived from undifferentiated gill precursor stem cells (Bols et al. 1994; Lee et al. 2009). The ion regulation of this cell line must thereby differ, at least to a certain degree, from the physiological functions described for rainbow trout gill cells. It can be reasonably expected that Na^+^/K^+^-ATPase is expressed in RTgill-W1 cells, as this pump is present in nearly all animal cells (Marshall 2002). Whether the functions of this pump are coupled with other ATPases relevant in *in-vivo* models remains to be elucidated. Clearly, in vivo as well as in vitro, PRMs seem to interfere with the Cl^−^ and Ca^2+^ homeostasis of the cells, ultimately leading to complete ionodysregulation should the toxin persist in the environment. It is interesting, how Na^+^ elimination from the cell culture medium had no effect on the PRM toxicity in both RTgill-W1 as well as in HCEC-1CT cells. Specifically, because Na^+^ and Cl^−^ regulation is often strongly interspersed (Hwang et al. 2011; Marshall 2002). Seeing how gills are vital organs for osmoregulation, the effect PRMs have on the ionoregulation is either a result of PRM toxicity or integral to the toxic mode of action or PRMs (Hwang et al. 2011; Lee et al. 2009). Further studies are needed to better understand this correlation.

In conclusion, it was shown that toxicity of *P. parvum* extracts is indeed caused by PRMs, although a combined effect with other unidentified compounds cannot be ruled out. Furthermore, the results clearly show that toxicity of PRMs is not limited to fish cells, but human cells as exemplified for HCEC-1CT, are affected in comparable concentration ranges. Strains producing the same type of PRM do not necessarily synthesize the same PRM analogs, and apparently, the kind and relative amount of analog used for toxicity testing impacts the overall toxicity. Studying the cytotoxicity of single analogs could paint a clearer picture of the mode of action of PRMs. Comparing the potency and the structure of each may reveal which structural and functional moieties are more prominent in highly toxic strains. Visible changes in the plasma membrane and nuclei resulted from exposure to PRMs, but so far it remains unclear how these ichthyotoxins induce that change. The effects in RTgill-W1 cells resulting from PRM exposure could be impacted by the absence of Cl^−^ and Ca^2+^ ions. It would be advisable to further investigate the impact of these ions on the PRM toxicity. For a complete picture, the role ion channels and regulators for osmotic balance play should be examined. As Cl^−^ lowered cytotoxic activity towards HCEC-1CT and in part towards RTgill-W1 cells, it would be of particular interest to study the involvement of Cl^−^-channels on a molecular level. Other membrane structures, such as cholesterol, sphingomyelins or phospholipids could also be relevant in PRM toxicity and interactions with those are worth exploring as well.

### Electronic Supplementary Material

Below is the link to the electronic supplementary material.

Supplementary material 1 (DOCX 15517 kb)

## Data Availability

The authors declare that the data supporting the findings of this study are available within the paper and its Supplementary Information files. Should any raw data files be needed in another format they are available from the corresponding author upon reasonable request.
