# Correction to: Cytotoxicity of *Prymnesium parvum* extracts and prymnesin analogs on epithelial fish gill cells RTgill-W1 and the human colon cell line HCEC-1CT

**DOI:** 10.1007/s00204-024-03716-3

**Published:** 2024-04-09

**Authors:** Elisabeth Varga, Hélène-Christine Prause, Matthias Riepl, Nadine Hochmayr, Deniz Berk, Eva Attakpah, Endre Kiss, Nikola Medić, Giorgia Del Favero, Thomas Ostenfeld Larsen, Per Juel Hansen, Doris Marko

**Affiliations:** 1 Department of Food Chemistry and Toxicology, Faculty of Chemistry, University of Vienna, Währinger Str. 38-40, 1090 Vienna, Austria; 2 Unit Food Hygiene and Technology, Institute of Food Safety, Food Technology and Veterinary Public Health, University of Veterinary Medicine, Vienna, Veterinärplatz 1, 1210 Vienna, Austria; 3 Vienna Doctoral School in Chemistry, Faculty of Chemistry, University of Vienna, Währinger Str. 42, 1090 Vienna, Austria; 4 Core Facility Multimodal Imaging, Faculty of Chemistry, University of Vienna, Währinger Str. 38-42, 1090 Vienna, Austria; 5 Marine Biological Section, Department of Biology, University of Copenhagen, Strandpromenaden 5, 3000 Helsingør, Denmark; 6 Center for Bioresources, Division for Food and Production, Danish Technological Institute, Gregersensvej 8, 2630 Taastrup, Denmark; 7 Department of Biotechnology and Biomedicine, Technical University of Denmark, Søltofts Plads 221, 2800 Kgs Lyngby, Denmark

**Correction to: Archives of Toxicology (2024) 98:999–1014** 10.1007/s00204-023-03663-5

In this article, the structures of yyyy two chlorine atoms were missing in Fig. 1 and should have been appeared as:

Incorrect version:
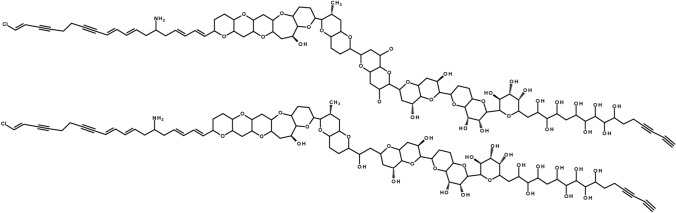


Corrected version:

**Fig. 1:**
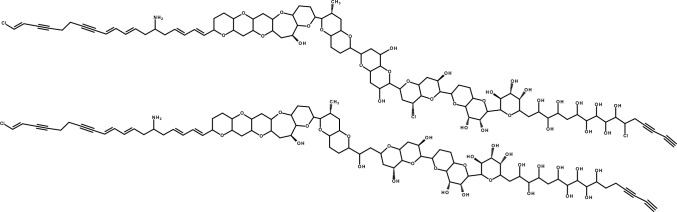
Backbone structure of **A**- (top) and **B**-type (bottom) prymnesins according to Igarashi et al. (1996) and Rasmussen et al. (2016a), respectively. The main difference is the length of the carbon backbone, further modifications of prymnesin analogs are attached sugar moieties, the degree of saturation and the number of incorporated chloride and oxygen atoms

