# Supplementary material for: Cytotoxicity of Prymnesium parvum extracts and prymnesin analogs on epithelial fish gill cells RTgill-W1 and the human colon cell line HCEC-1CT
Source: Arch Toxicol. 2024 Jan 11;98(3):999–1014. doi: 10.1007/s00204-023-03663-5 (PMC10861388; doi:10.1007/s00204-023-03663-5)
Supplement: Supplementary file 1 — Supplementary material 1 (DOCX 15517 kb) [file 204_2023_3663_MOESM1_ESM.docx]

**Cytotoxicity of *Prymnesium parvum* extracts and prymnesin analogs
on epithelial fish gill cells RTgill-W1 and the human colon cell line HCEC-1CT**

Elisabeth Varga^1,2,#,^* and Hélène-Christine Prause^1,3,#^, Matthias Riepl^1^, Nadine Hochmayr^1^, Deniz Berk^1^, Eva Attakpah^1^, Endre Kiss^1,4^, Nikola Medić^5,6^, Giorgia Del Favero^1,4^, Thomas Ostenfeld Larsen^7^, Per Juel Hansen^5^, Doris Marko^1^

1 Department of Food Chemistry and Toxicology, Faculty of Chemistry, University of Vienna, Währinger Str. 38-40, 1090 Vienna, Austria.

2 Unit Food Hygiene and Technology, Institute of Food Safety, Food Technology and Veterinary Public Health, University of Veterinary Medicine, Vienna, Veterinärplatz 1, 1210 Vienna, Austria.

3 Vienna Doctoral School in Chemistry, Faculty of Chemistry, University of Vienna, Währinger Str. 42, 1090 Vienna, Austria.

4 Core Facility Multimodal Imaging, Faculty of Chemistry, University of Vienna, Währinger Str. 38-42, 1090 Vienna, Austria.

5 Marine Biological Section, Department of Biology, University of Copenhagen, Strandpromenaden 5, 3000 Helsingør, Denmark.

6 Center for Bioresources, Division for Food and Production, Danish Technological Institute, Gregersensvej 8, 2630 Taastrup, Denmark

7 Department of Biotechnology and Biomedicine, Technical University of Denmark, Søltofts Plads 221, 2800 Kgs. Lyngby, Denmark.

# Both authors contributed equally.

*Correspondence: elisabeth.varga@vetmeduni.ac.at; Tel.: +43 1 25077-3302

SI Table 1 Information on main differences of two RTgill-W1 cultivation media.

| **L-15 complete media** | **Leibovitz’s 15 media information** | | **Complemented with (%)** | | |
| --- | --- | --- | --- | --- | --- |
|  | **Reference and manufacturer** | **Phenol red** | **L-glutamine (*v/v*)** | **FCS^#^ (v/v)** | **P/S^#^ (v/v)** |
| **Recipe 1** | 21083027, Thermo Fisher Scientific | no | 1 (included) | 10 | 1 |
| **Recipe 2** | L5520, Sigma-Aldrich | yes | 1* | 10 | 1 |

FCS Fetal calf serum; P/S; penicillin/streptomycin

*added separately from Thermo Fisher Scientific, Waltham, MA, USA

^#^Thermo Fisher Scientific, Waltham, MA, USA

SI Table 2 Media composition of ion-free media and media used for live-cell imaging.

| *Media for HCEC-1CT cells* | *Desired concentration (mM)* | *Media for RTgill-W1 cells* | *Desired concentration (mM)* |
| --- | --- | --- | --- |
| Normal External Solution (NES) |  | **NES** |  |
| NaCl | 140.0 | NaCl | 140.0 |
| KCl | 2.8 | KCl | 5.6 |
| CaCl_2_ | 2.0 | CaCl_2_ | 1.3 |
| MgCl_2_ | 2.0 | MgCl_2_ | 1.7 |
| HEPES | 10.0 | HEPES | 10.0 |
| Glucose | 10.0 | Galactose | 5.0 |
| pH adjusted to 7.3 with NaOH |  | pH adjusted to 7.3 with NaOH |  |
| Na^+^ free medium |  | **Na^+^ free medium** |  |
| N-methyl-D-glucamine | 140.0 | N-methyl-D-glucamine | 140.0 |
| KCl | 2.8 | KCl | 5.6 |
| CaCl_2_ | 2.0 | CaCl_2_ | 1.3 |
| MgCl_2_ | 2.0 | MgCl_2_ | 1.7 |
| HEPES | 10.0 | HEPES | 10.0 |
| Glucose | 10.0 | Galactose | 5.0 |
| pH adjusted to 7.3 with KOH |  | pH adjusted to 7.3 with KOH |  |
| Ca^2+^ free medium |  | **Ca^2+^ free medium** |  |
| NaCl | 140.0 | NaCl | 140.0 |
| KCl | 2.8 | KCl | 5.6 |
| MgCl_2_ | 5.0 | MgCl_2_ | 1.7 |
| EGTA | 2.0 | EGTA | / |
| HEPES | 10.0 | HEPES | 10.0 |
| Glucose | 10.0 | Galactose | 5.0 |
| pH adjusted to 7.3 with NaOH |  | pH adjusted to 7.3 with NaOH |  |
| Cl^-^ free medium |  | **Cl^-^ free medium** |  |
| Na_2_SO_4_ | 140.0 | Na-acetate | 140.0 |
| K-gluconate | 2.5 | K-gluconate | 17.4 |
| MgSO_4_ x 7 H_2_O | 1.0 | Mg(CH_3_COO)_2_ x 4 H_2_O | 3.7 |
| EGTA | 2.0 | HEPES | 10.0 |
| CaSO_4_ x 2 H_2_O | 2.0 | CaSO_4_ x 2 H_2_O | 2.0 |
| Glucose | 10.0 | Galactose | 5.0 |
|  |  | Na-pyruvate | 50.0 |
| pH adjusted to 7.3 with NaOH |  | pH adjusted to 7.3 with NaOH |  |

**SI Table 3** Description and composition of all used samples, including some single compound prymnesin (PRM) solutions. The identified analogs in the extracts are provided in SI Table 4. EC_50_ values were translated to the molar sum of all PRMs present, calculated using formula 1.

| **Strain** | **ID** | **Characteristics** | **Stock concentration** |  | | **EC_50_** | | |
| --- | --- | --- | --- | --- | --- | --- | --- | --- |
|  |  | **Analogs identified via UHPLC/HRMS** | **Semi-quantified (µM)** | **RTgill-W1** | | | **HCEC-1CT** | |
|  |  |  |  | **(nM)** | **Sum_PRM_ (µg/L) ^1^** | | **(nM)** | **Sum_PRM_ (µg/L) ^1)^** |
| **UTEX-2797** | A1 | A-type biomass extract | 20 ± 2 | / |  | | 12.7 ± 0.3 | 26 ± <1 |
|  | A2 | A-type biomass extract | 20 ± 2 | 10.3± 0.3 | 20 ± <1 | | 22.1 ± 5.6 | 43 ± 11 |
|  | sA1 | PRM-A (3Cl) + pentose  (purified) | 58 ± 6 | / |  | | 76 ± 34 | 149 ± 65 |
| **n.d. (Sigma Aldrich)** | A3 | mixture of A-type prymnesins | 3.6 ± 0.3 | 4.0 ± 0.2 | 8.7 ± 0.4 | | 6.2 ± 0.1 | 13 ± <1 |
| **K-0081** | B1 | B-type biomass extract | 348 ± 39 | / |  | | 170 ± 9 | 307 ± 16 |
|  | B2 | B-type biomass extract | 145 ± 15 | / |  | | 285 ± 21 | 506 ±37 |
|  | B3 | B-type biomass extract | 204 ± 13/ 139 ± 18 | 110 ± 11 | 200 ± 20 | | 258 ± 126 | 460 ± 230 |
|  | B4 | B-type biomass extract | 393 ± 50 | / |  | | 300* | 560* |
|  | sB1 | PRM-B (1 Cl) + pentose; PRM-B (1 Cl) + hexose  (purified) | 92 ± 9 | / |  | | 220 ± 30 | 395 ± 55 |
|  | sB2 | PRM-B (1Cl) + 2 hexose; PRM-B (1Cl) hexose + pentose (purified) | 60 ± 7 | / |  | | 270 ± 160 | 527 ± 312 |
| **RCC-1436** | C1 | C-type biomass extract | 17 ± 2 | 13.9 ± 0.4 | 26.0 ± 0.7 | | ca 14** | ca 26** |
| **RCC-191** | C2 | C-type biomass extract | 40 ± 8 | 9.8 ± 0.8 | 19.3 ± 1.6 | | ca 33** | ca 65** |

***/*** not conducted

* no standard deviation is provided since the EC_50_-value is based on only one biological replicate

** not possible to calculate an EC_50_-value with Rstudio since 0% metabolic activity is not reached and a high variability is observed around the EC_50_-value

SI Table 4 Prymnesin (PRM) composition of Prymnesium parvum extracts in %. Analogs were identified via UHPLC/MS.

| *A-type PRM* | *UTEX-2797* | | | *Sigma solution* |
| --- | --- | --- | --- | --- |
|  | **A1** | **A2** | **sA1** | **A3** |
| PRM-A (2 Cl) | 2 | - | - | - |
| PRM-A (2 Cl + DB) | 1 | - | - | - |
| PRM-A (2 Cl) + pentose | 2 | - | - | 2 |
| PRM-A (2 Cl + DB) + pentose | 17 | 5 | - | - |
| PRM-A (2 Cl) + 2 pentose + hexose | - | - | - | 4 |
| PRM-A (3Cl) | 5 | 8 | 8 | - |
| PRM-A (3 Cl) + pentose | 41 | 81 | 92 | 18 |
| PRM-A (3 Cl) + pentose + hexose | - | - | - | 9 |
| PRM-A (3 Cl) + hexose | 1 | - | - | - |
| PRM-A (3 Cl) + 2 pentose | 2 | - | - | 3 |
| PRM-A (3 Cl) + 2 hexose | 6 | - | - | - |
| PRM-A (3 Cl) + 2 pentose + hexose | 23 | 6 | - | 63 |

| *B-type PRM* | *K-0081* | | | | | |
| --- | --- | --- | --- | --- | --- | --- |
|  | **B1** | **B2** | **B3** | **B4** | **sB1** | **sB2** |
| PRM-B (1 Cl) | 21 | 22 | 24 | 8 | - | - |
| PRM-B (1 Cl) + pentose | 17 | 19 | 18 | 9 | 87 | - |
| PRM-B (1 Cl) + hexose | 52 | 42 | 41 | 52 | 13 | 16 |
| PRM-B (1 Cl) + pentose + hexose | 1 | 2 | 2 | 2 | - | 5 |
| PRM-B (1 Cl) + 2 hexose | 5 | 9 | 10 | 25 | - | 79 |
| PRM-B (2 Cl) | 1 | 1 | 1 | - | - | - |
| PRM-B (2 Cl) + pentose | 1 | 1 | 1 | 4 | - | - |
| PRM-B (2 Cl) + hexose | 2 | 2 | 2 | 1 | - | - |
| PRM-B (2 Cl) + 2 hexose | < 1 | 1 | 1 | 1 | - | - |

| *C-type PRM* | *RCC-1436* | *RCC-191* |
| --- | --- | --- |
|  | **C1** | **C2** |
| PRM-C (2 Cl + DB) + pentose | 4 | - |
| PRM-C (2 Cl) + pentose + 2 hexose | - | * |
| PRM-C (3 Cl) | - | 5 |
| PRM-C (3 Cl + DB) | 8 | 1 |
| PRM-C (3 Cl + DB) + pentose | 17 | 5 |
| PRM-C (3 Cl + DB) + pentose + hexose | 1 | 2 |
| PRM-C (3 Cl + DB) + 2 pentose + hexose | - | 2 |
| PRM-C (3 Cl) + pentose | 8 | 12 |
| PRM-C (3 Cl) + 2 pentose + hexose | - | 2 |
| PRM-C (4 Cl + DB) | 28 | 14 |
| PRM-C (4 Cl + DB) + pentose | 36 | 42 |
| PRM-C (4 Cl + DB) + pentose + hexose | - | 8 |
| PRM-C (4 Cl + DB) + 2 pentose + hexose | - | 4 |
| PRM-C (4 Cl + 3 =O) | - | 1 |
| PRM-C (4 Cl + 3 =O) + pentose | - | 2 |

“-“ not detected; DB double bond; =O additional ketogroup;

* traces of PRM-C (2 Cl) + pentose + 2 hexose with the ion [M + Na + H]^+2^ can neither be excluded nor confirmed due to overlay with PRM-C (3 Cl + DB) + hexose + 2 pentose with the ion [M + NH_4_ + H]^+2^ (*m/z* 1086.4)

SI Table 5 Effective concentration 50 (EC_50_) values in nM for Prymnesium parvum extracts in RTgill-W1 cells after a 3 h and 24 h incubation period using the L-15 complete culture medium according to recipe 2.

| **Prymnesin type** | **Strain** | **ID** | **3 hours** | **24 hours** | **Factor difference** |
| --- | --- | --- | --- | --- | --- |
| A-type | Unknown (Sigma) | A3 | 4.0 ± 0.2 | 2.4 ± 0.2 | 1.7 |
| B-type | K-0081 | B1 | 110 ± 11 | 41.5 ± 0.6 | 2.6 |
| C-type | RCC-191 | C2 | 9.8 ± 0.8 | 4.5 ± 0.1 | 2.2 |

SI Table 6 Mean cellular area of HCEC-1CT cells per well in % after 1.5 hours (t_1_) and 3-hours (t_2_) of incubation with K-0081 extract B1. All values are related to the control (live cell imaging solution (LCIS)) before the incubation (t_0_) set to 100%. Data are provide as the mean of n=2. NES: normal external solution.

| **t_1_** | **LCIS** | **Cl^-^ free** | **Na^+^ free** | **Ca^2+^ free** | **NES** |
| --- | --- | --- | --- | --- | --- |
| Solvent | 100 | 111 | 106 | 106 | 105 |
| 165 nM | 91 | 104 | 74 | 89 | 76 |
| 325 nM | 96 | 106 | 79 | 95 | 77 |
| 645 nM | 84 | 103 | 78 | 89 | 76 |
| **t_2_** | **LCIS** | **Cl^-^ free** | **Na^+^ free** | **Ca^2+^ free** | **NES** |
| Solvent | 96 | 114 | 104 | 105 | 104 |
| 165 nM | 78 | 105 | 66 | 84 | 67 |
| 325 nM | 92 | 107 | 74 | 89 | 71 |
| 645 nM | 77 | 102 | 70 | 78 | 65 |

SI Table 7 Cytotoxic potential of B-type prymnesin extract B2 assessed in the HCEC-1CT complete medium (complete medium), live cell imaging solution (LCIS) and chlorine free medium after an incubation time of 3 hours. In case of the lactate dehydrogenase (LDH) assay the results are provided in percent relative to the LDH maximum control (treatment with lysis buffer) and the positive control (PC) was provided with the assay kit. In case of crystal violet (CV) the results are provided as cell viability in percent and are related to the respective solvent control (0.5 % ethanol). As positive control (PC), 0.075% (HCEC-1CT complete medium) or 0.05 % Triton X-100 (LCIS and Cl^-^ free medium) was applied. The data are shown as means ± standard deviations of at least three biological replicates, each performed in technical triplicates. The Shapiro-Wilk-Test was used for testing normality. Significant differences (* = p < 0.05) were calculated by One Way ANOVA, followed by the posthoc Fisher´s least significant difference (LSD) test.

|  | **LDH** | | | **CV** | | |
| --- | --- | --- | --- | --- | --- | --- |
| **Extract B2** | **complete medium** | **LCIS** | **Cl^-^ free** | **complete medium** | **LCIS** | **Cl^-^ free** |
| 80 nM | 0 ± 1 % | 1 ± 1 % | 1 ± 1 % | 113 ± 9 % | 119 ± 25 % | 109 ± 10 % |
| 325 nM | 1 ± 2 % | * 7 ± 1 % | 0 ± 1 % | * 75 ± 14 % | 90 ± 16 % | 111 ± 11 % |
| 645 nM | * 71 ± 1 % | * 8 ± 2 % | 2 ± 1 % | * 19 ± 4 % | 92 ± 16 % | 97 ± 5 % |
| PC | 28 ± 2 % | 39 ± 5 % | 34 ± 6 % | 54 ± 13 % | 80 ± 15 % | 75 ± 13 % |

$$PRManalog \left( \frac{\mu g}{L} \right)=\frac{\left( \left( EC50 \left( \frac{nmol}{L} \right)*\% PRManalog \right)*M PRManalog(\frac{ng}{nmol}) \right)}{1000}$$

SI Formula 1 Calculation for the sum of prymnesins (PRMs) in the single substance solutions based on their molecular weight

B

A

C

**
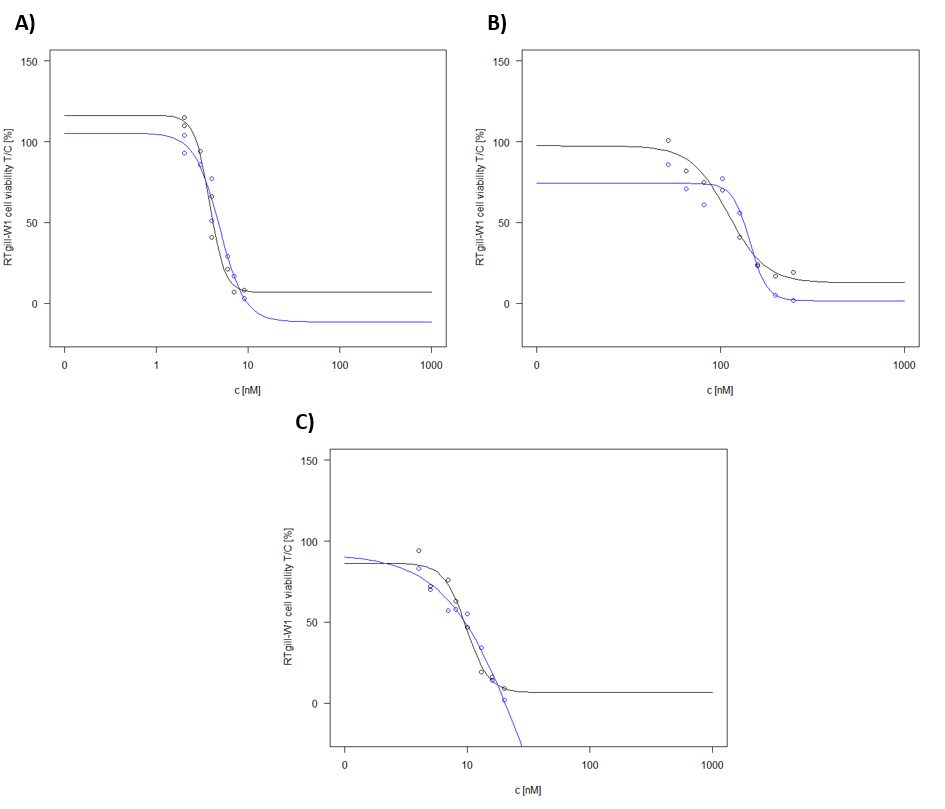
**

SI Fig. 1 Cytotoxic potency of prymnesins (PRM) in two RTgill-W1 culture media compared after a 3-hour exposure. The blue line represents the results in medium according to recipe 1 and the black line represents those in medium according to recipe 2. Dose response-curves are provided for the A-type PRM solution A3 (A), B shows the B-type PRM extract B3 from strain K-0081, and the C-type prymnesin extract C2 from strain RCC-191 is shown in C.


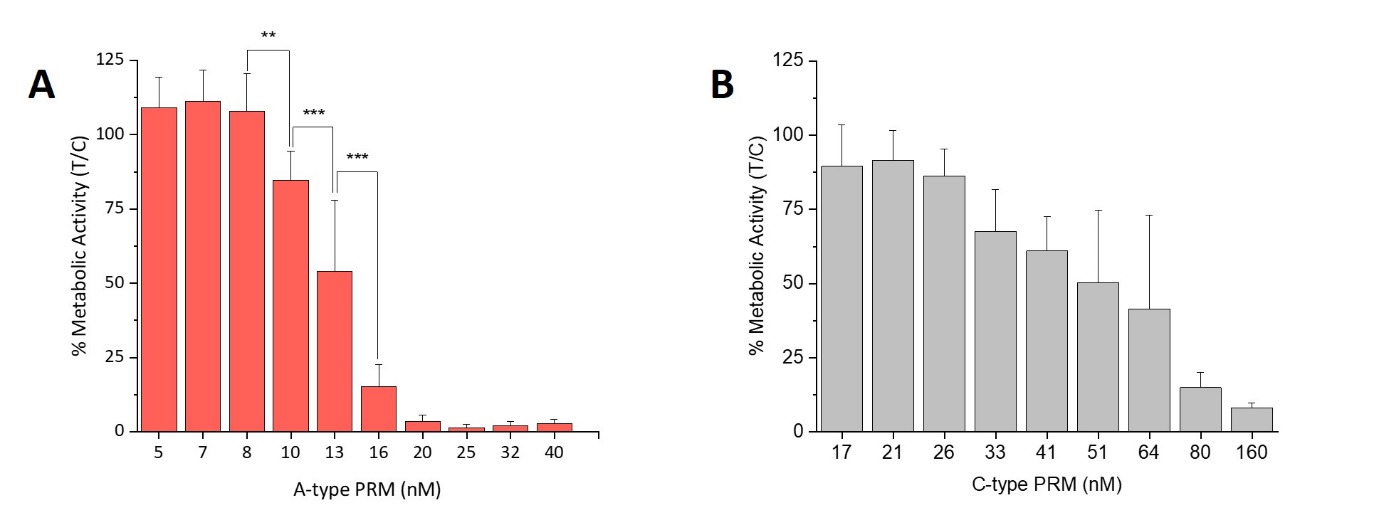


SI Fig. 2 Cell viability and membrane integrity of HCEC-1CT cells after 3-hour incubation with A-type prymnesin (PRM) extracts A1 obtained from the UTEX-2797 strain (A). Data are represented as mean ± SD of n = 4 for CellTiter blue (CTB), and n= 3 for data obtained through lactate dehydrogenase (LDH) measurements (dotted line). CTB results for C-type the PRM extract from RCC-191 are shown in B as mean ± SD of n ≥ 3.


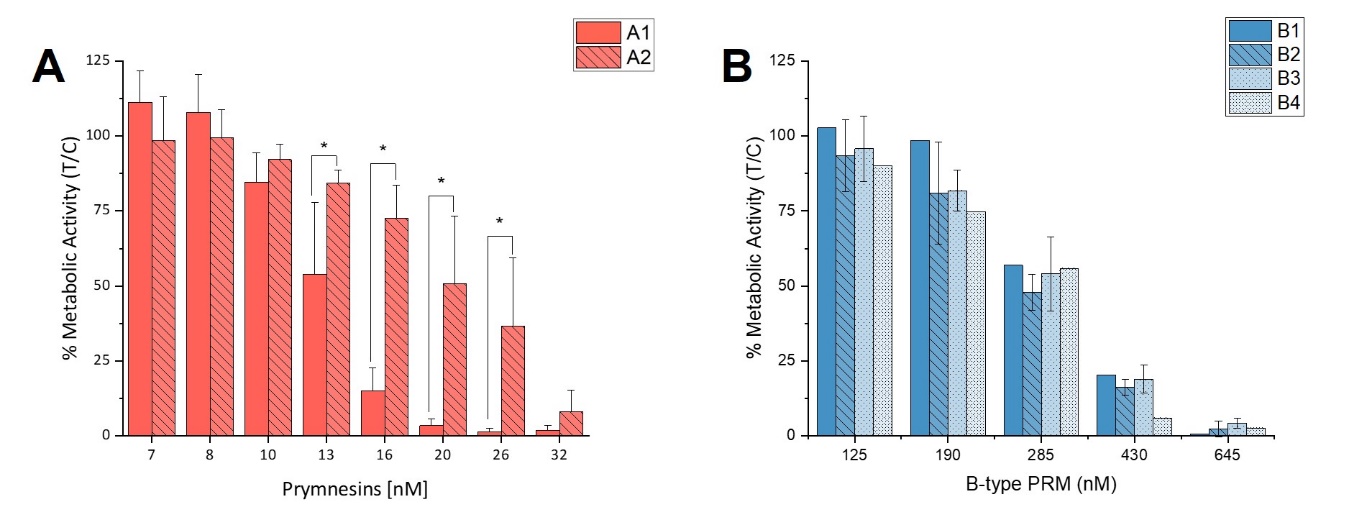


SI Fig. 3 Comparison between prymnesin (PRM) extracts taken from of the same Prymnesium parvum strain culture at different times. Data show the cell viability of HCEC-1CT cells after exposure to A UTEX-2797 PRM samples A1 and A2 and B to K-0081 PRM samples B1, B2, B3, and B4. Data show mean ± SD of n ≥ 3. If no standard deviation is displayed (B1 und B4) only one biological replicate was performed. Please notice that in case of B1 further replicates were tested but applying different dilutions and hence concentrations (see Fig. 3 of the main manuscript).


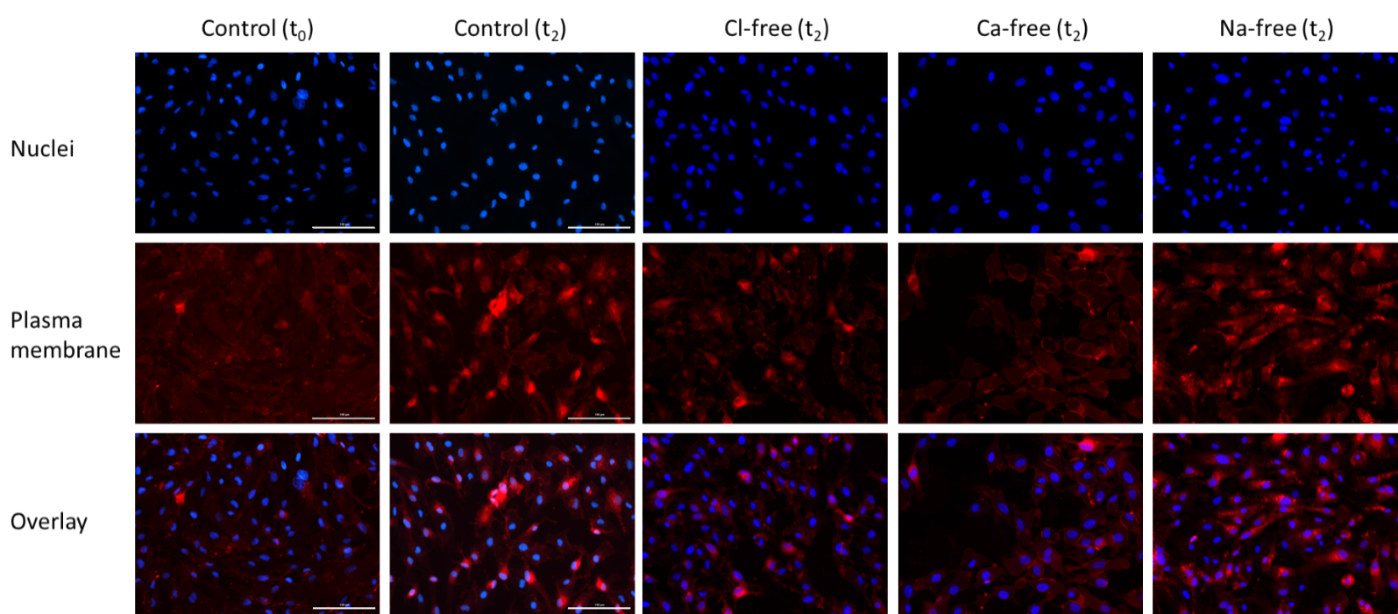


SI Fig. 4 Live cell images of RTgill-W1 cells in the control medium (normal external solution (NES)) before (t_0_) and after a 3-hour (t_2_) exposure to the A-type prymnesin (PRM) sample A3 in different media. Scale bar measures 100 µm. Brightness has been adjusted for images of the control for better visualization.


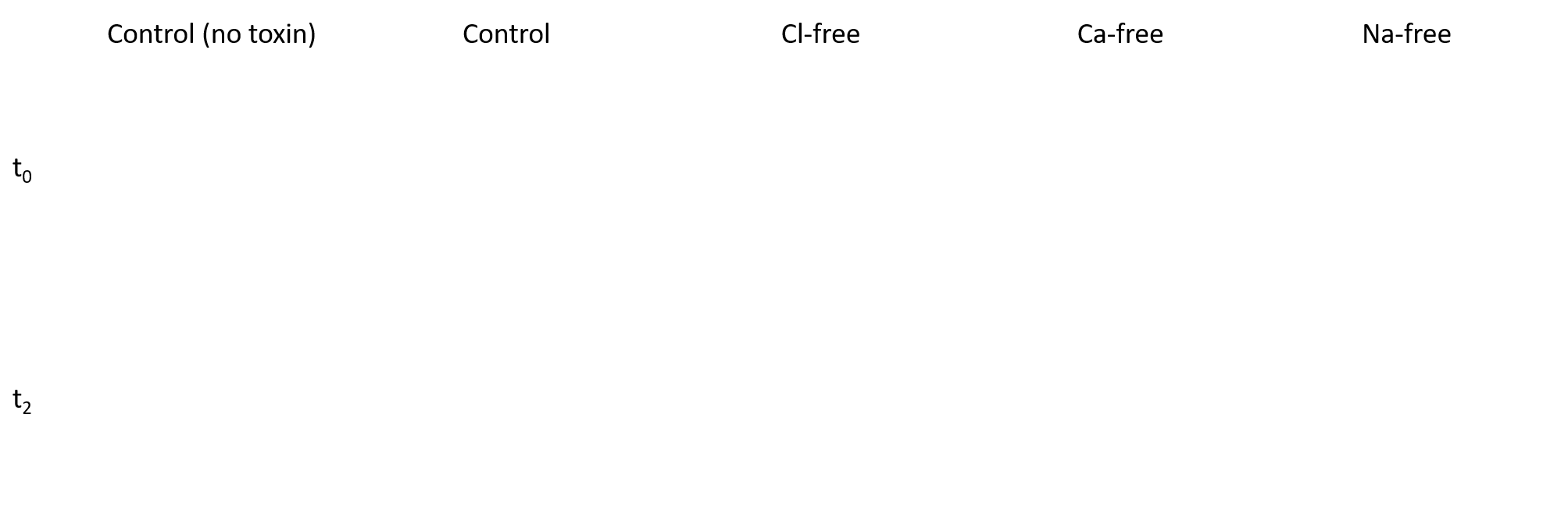

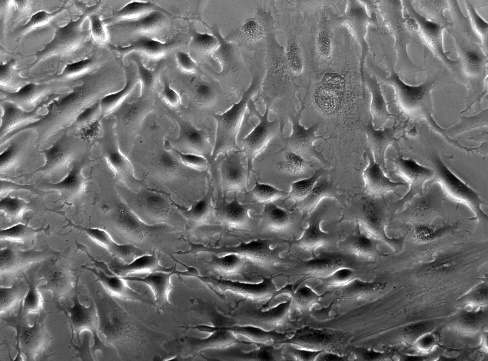

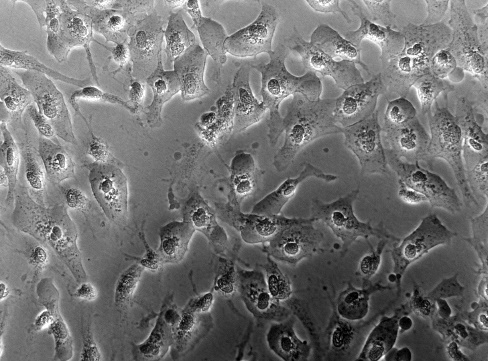

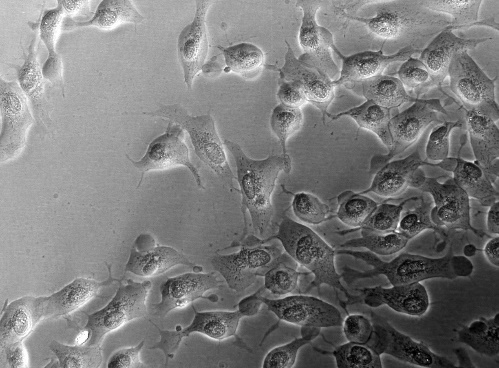

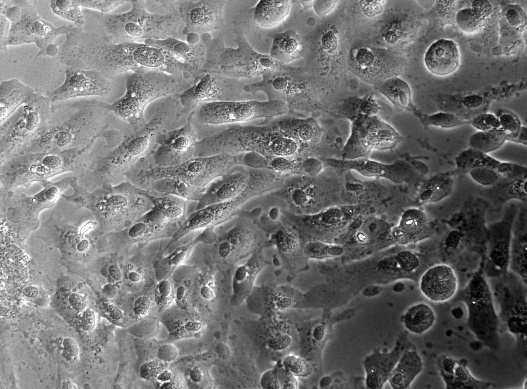

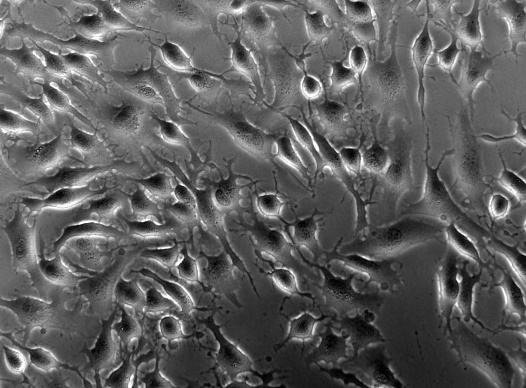

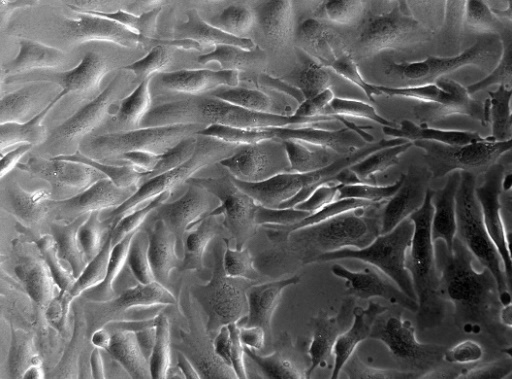

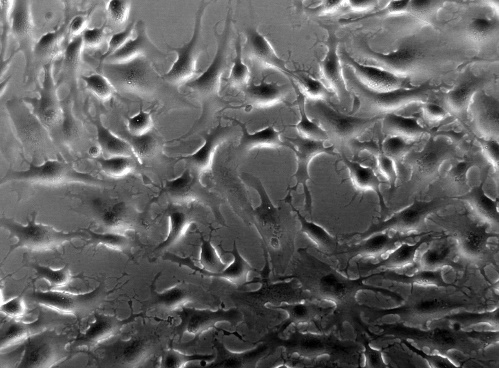

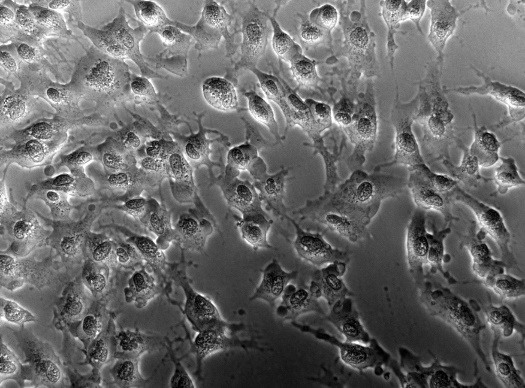

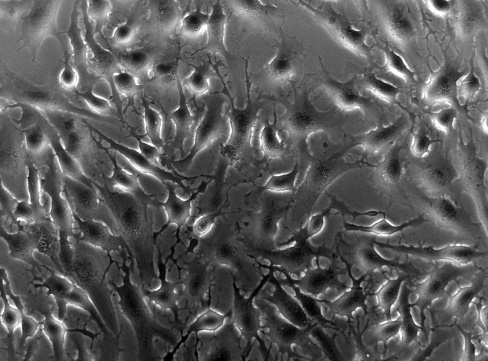

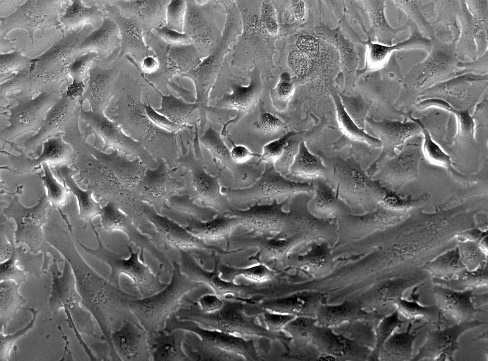


SI Fig. 5 Phase contrast images of RTgill-W1 cells before and after 3-hour exposure to the A-type prymnesin (PRM) sample A3 in different ion-free media. Scale bar represents 100 µm.

SI Fig. 6 Ratio of the cellular area of manually selected RTgill-W1 cells after exposure to prymnesins (PRMs) to the corresponding area of the cell nuclei. Data are provided as mean ± SD of n ≥ 36.


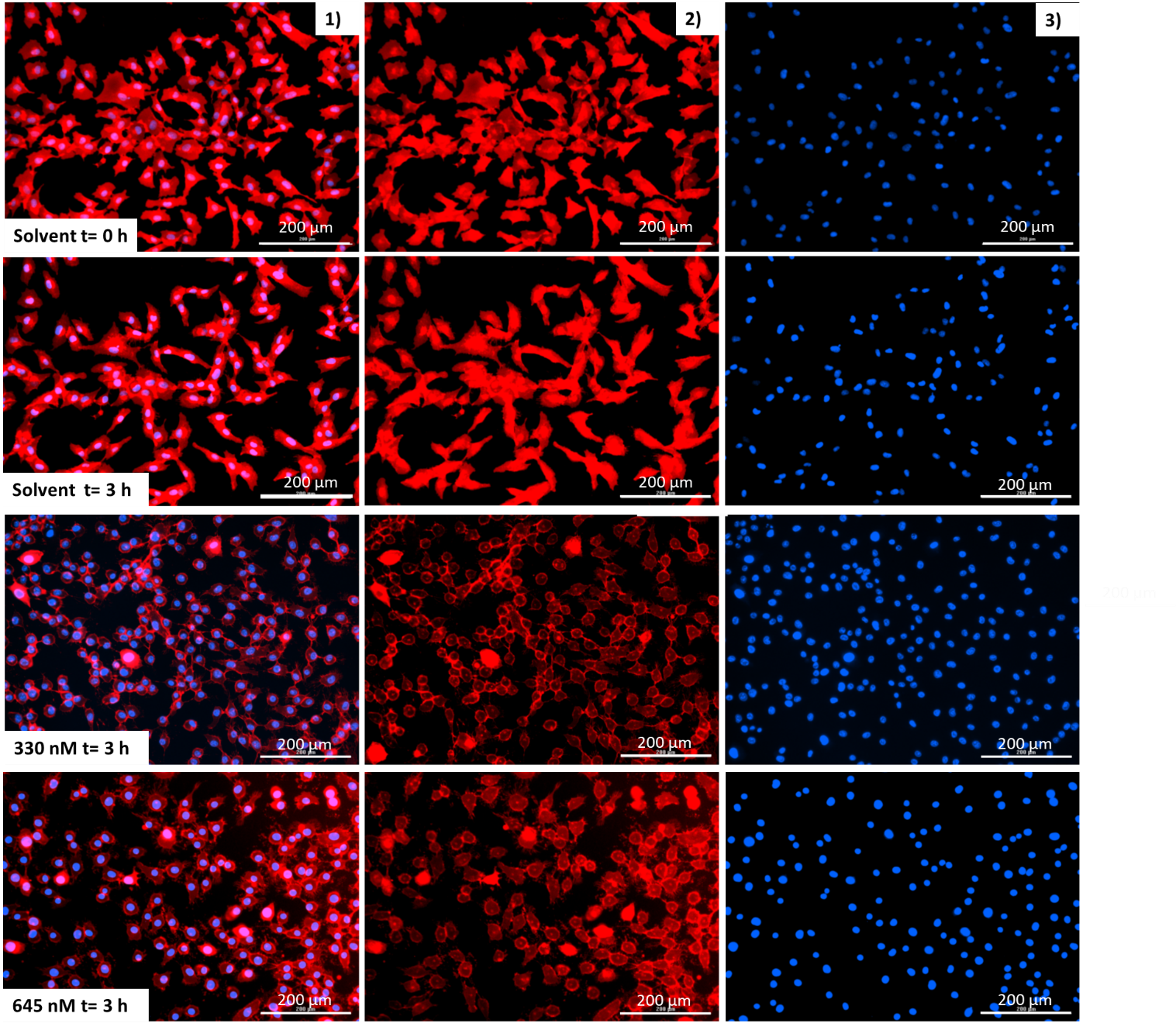


SI Fig. 7 Live cell images of HCEC-1CT cells treated with a K-0081 extract (extract B2) in live cell imaging solution. The figure illustrates the cell morphology at the very start of the incubation period and after a 3-hour treatment with the solvent control (0.5% ethanol) or the toxic extract respectively. In column 1), the overlay of cell mask and cell nuclei are shown. The cell membrane staining is provided in column 2) and the cell nuclei in column 3).


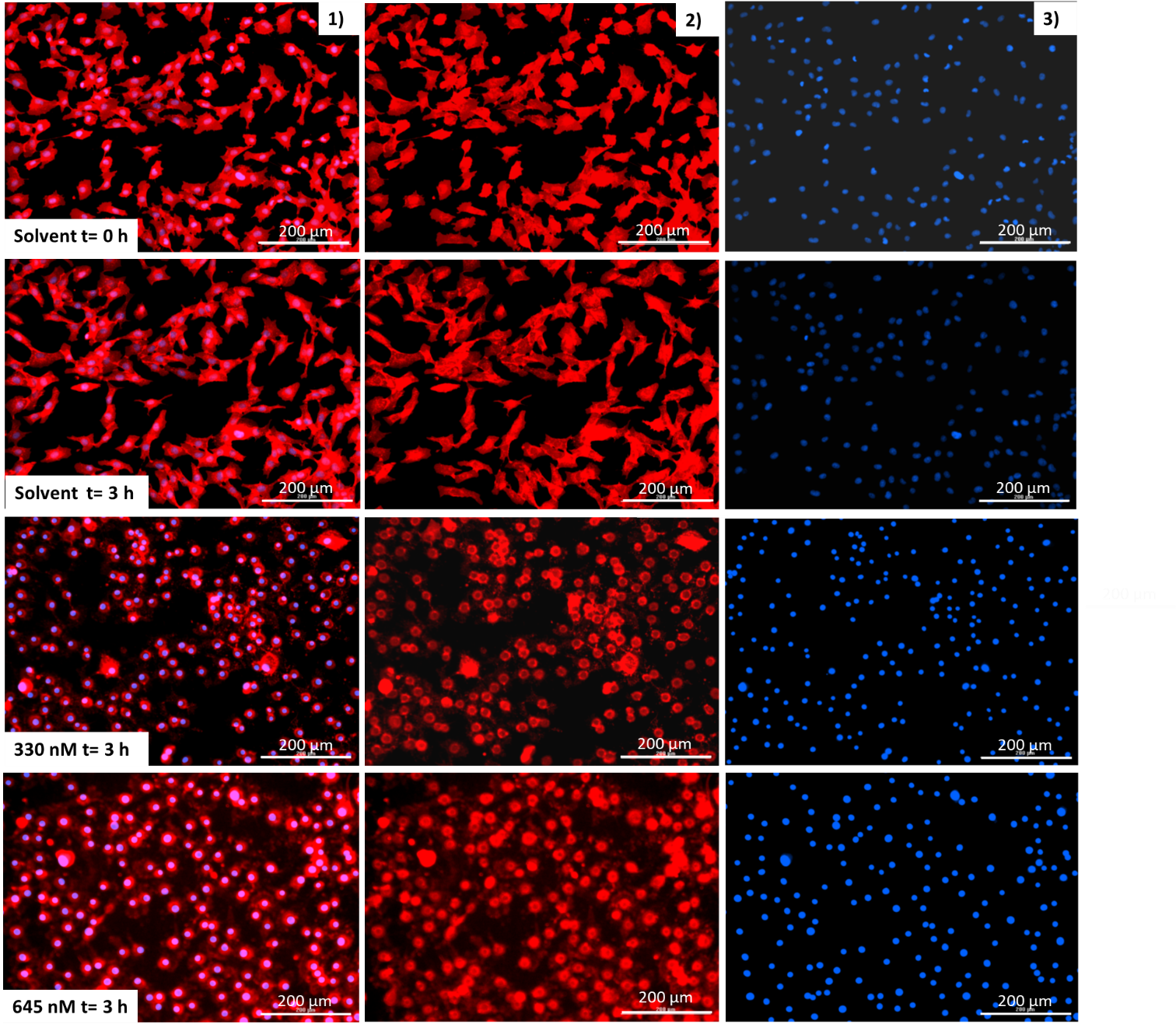


SI Fig. 8 Live cell images of HCEC-1CT cells treated with a K-0081 extract (extract B2) in Na^+^ free medium. The figure illustrates the cell morphology at the very start of the incubation period and after a 3-hour treatment with the solvent control (0.5% ethanol) or the toxic extract, respectively. In column 1), the overlay of cell mask and cell nuclei are shown. The cell membrane staining is provided in column 2) and the cell nuclei in column 3).


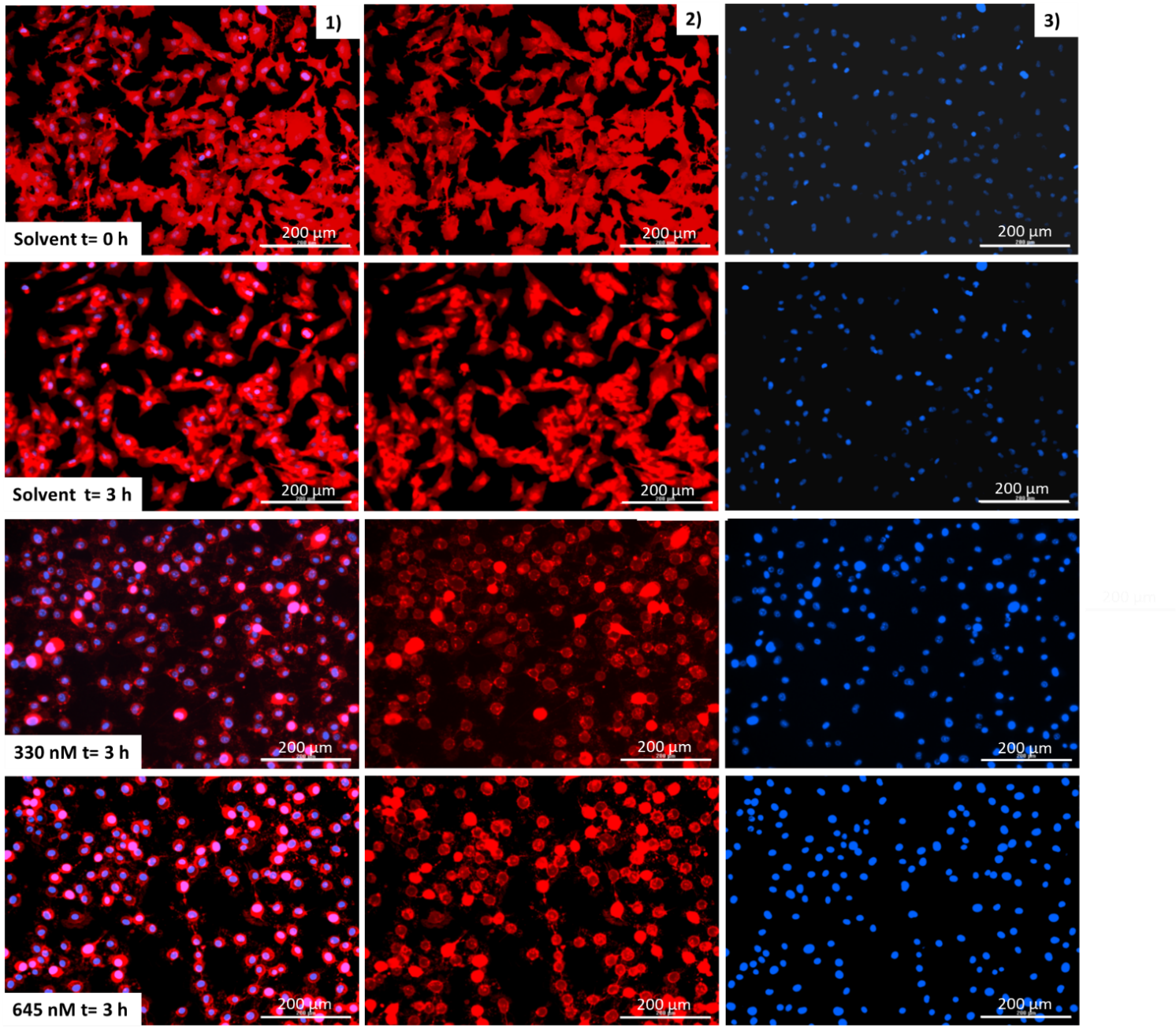


SI Fig. 9 Live cell images of HCEC-1CT cells treated with a K-0081 extract (extract B2) in Ca^2+^ free medium. The figure illustrates the cell morphology at the very start of the incubation period and after a 3-hour treatment with the solvent control (0.5% ethanol) or the toxic extract, respectively. In column 1), the overlay of cell mask and cell nuclei are shown. The cell membrane staining is provided in column 2) and the cell nuclei in column 3).


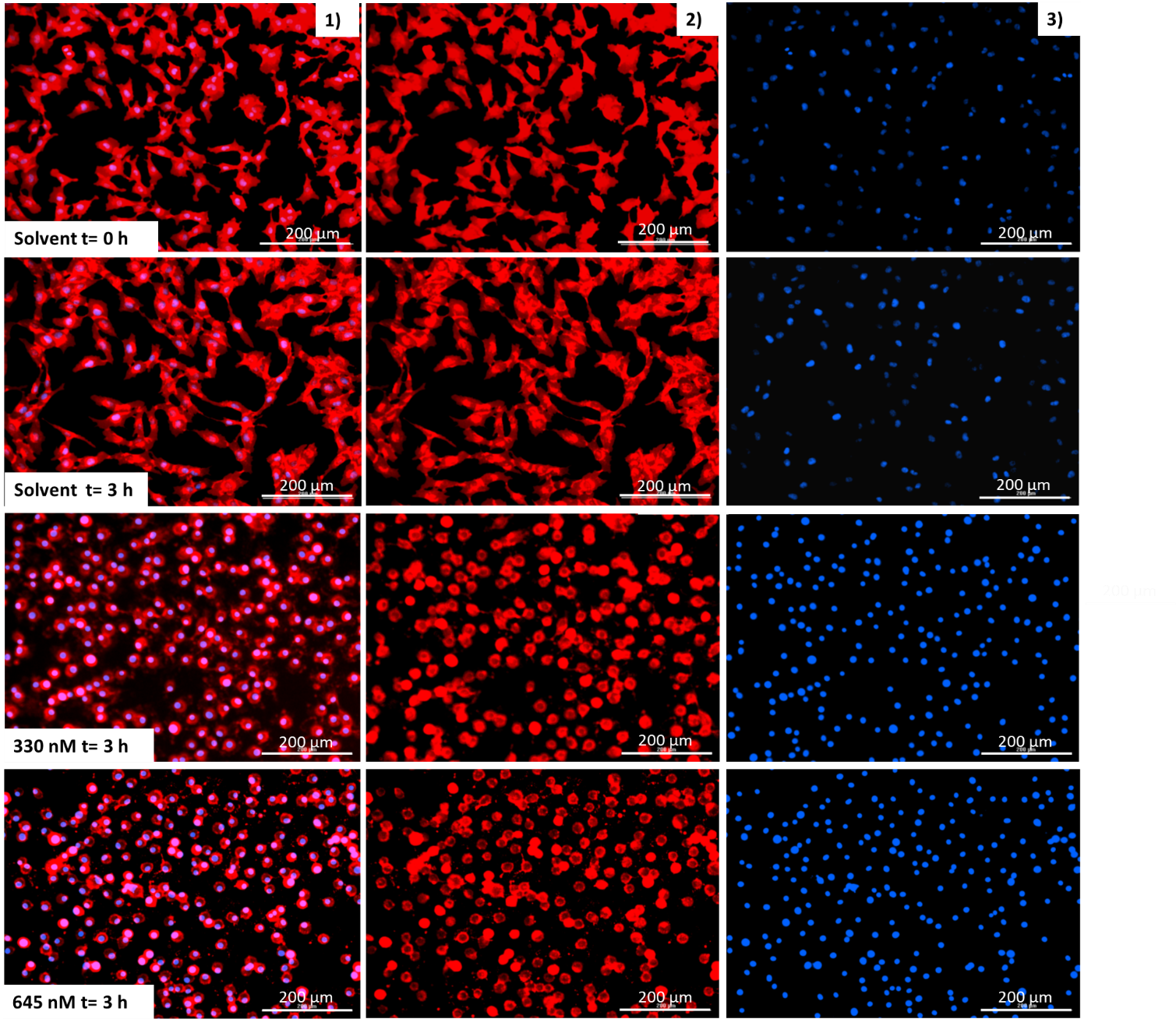


SI Fig. 10 Live cell images of HCEC-1CT cells treated with a K-0081 extract (extract B2) in normal external solution. The figure illustrates the cell morphology at the very start of the incubation period and after a 3-hour treatment with the solvent control (0.5% ethanol) or the toxic extract, respectively. In column 1), the overlay of cell mask and cell nuclei are shown. The cell membrane staining is provided in column 2) and the cell nuclei in column 3).


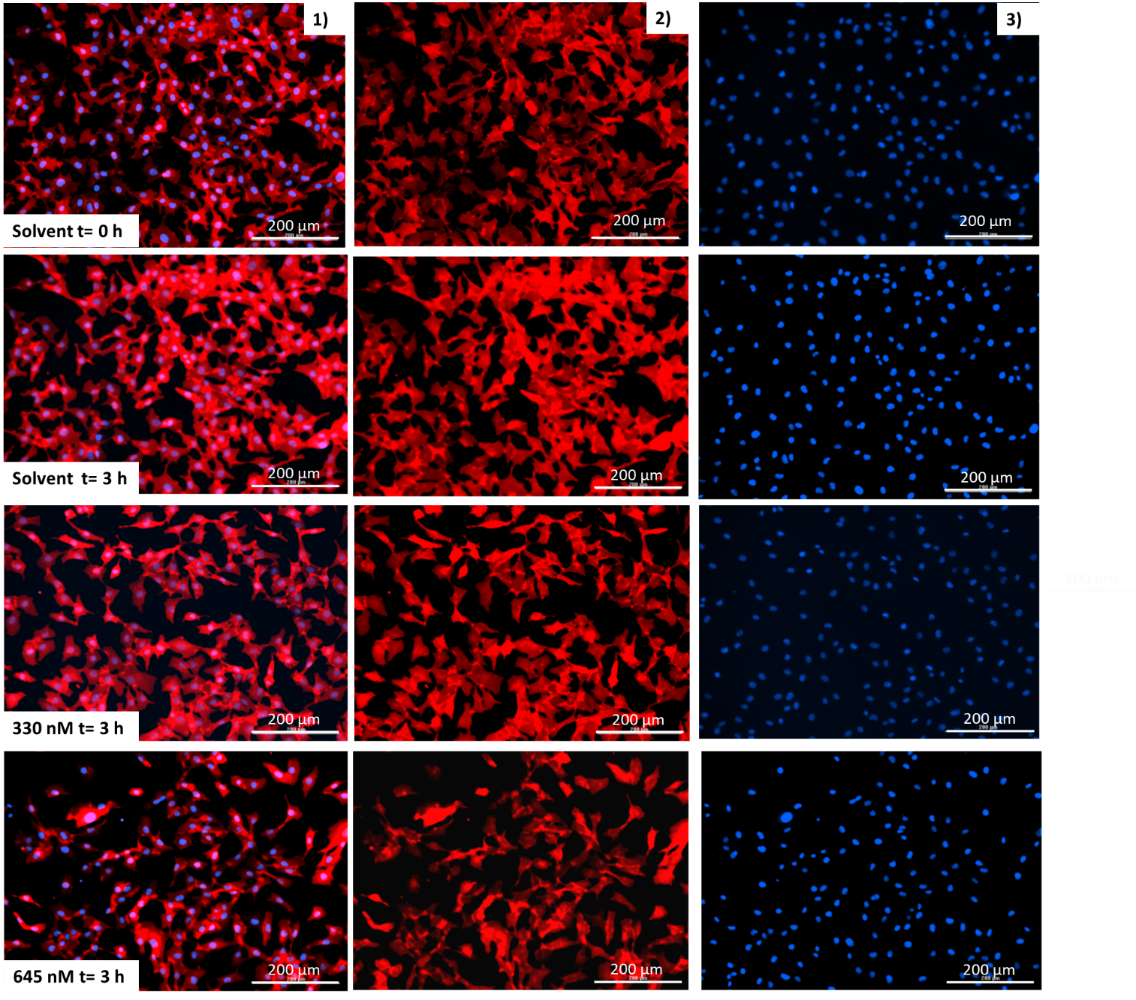


SI Fig. 11 Live cell images of HCEC-1CT cells treated with a K-0081 extract (extract B2) in Cl^-^-free medium. The figure illustrates the cell morphology at the very start of the incubation period and after a 3-hour treatment with the solvent control (0.5% ethanol) or the toxic extract, respectively. In column 1), the overlay of cell mask and cell nuclei are shown. The cell membrane staining is provided in column 2) and the cell nuclei in column 3).


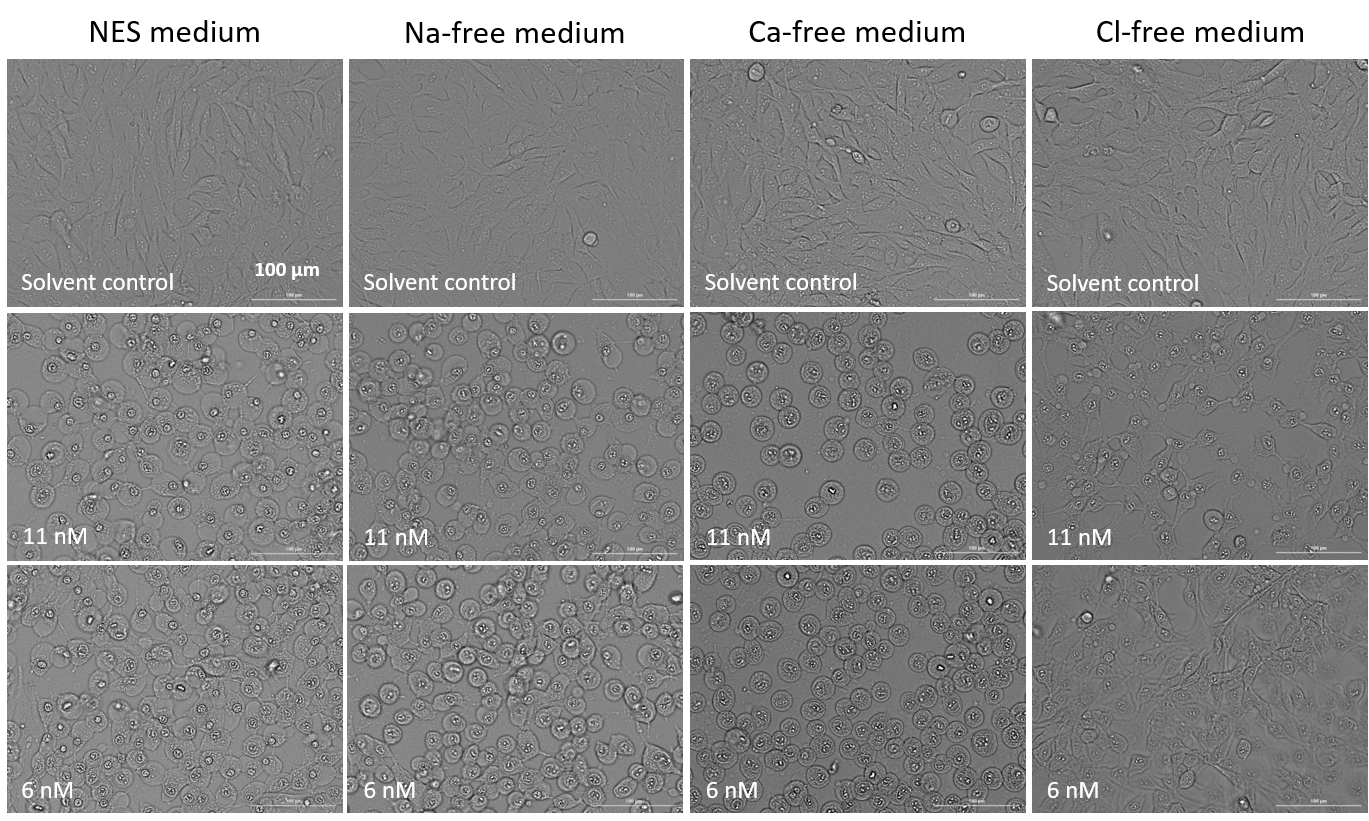


SI Fig. 12 Bright field images of HCEC-1CT cells after 3-hour exposure to A-type prymnesin solution sA1. Bars represent 100 µm.
